# Nanoscale fluorescence imaging of biological ultrastructure via molecular anchoring and physical expansion

**DOI:** 10.1186/s40580-022-00318-6

**Published:** 2022-07-09

**Authors:** Wei Wang, Yat Ho Chan, SoYoung Kwon, Jamuna Tandukar, Ruixuan Gao

**Affiliations:** 1 Department of Chemistry, College of Liberal Arts and Sciences, University of Illinois Chicago, Chicago, IL USA; 2 Department of Biomedical and Health Information Sciences, College of Applied Health Sciences, University of Illinois Chicago, Chicago, IL USA; 3 Department of Biological Sciences, College of Liberal Arts and Sciences, University of Illinois Chicago, Chicago, IL USA

**Keywords:** Bioimaging, Fluorescence imaging, Nanoscale, Nanoimaging, Super-resolution microscopy, Hydrogel, Tissue clearing, Tissue expansion, Spatial omics, Expansion microscopy

## Abstract

Nanoscale imaging of biological samples can provide rich morphological and mechanistic information about biological functions and dysfunctions at the subcellular and molecular level. Expansion microscopy (ExM) is a recently developed nanoscale fluorescence imaging method that takes advantage of physical enlargement of biological samples. In ExM, preserved cells and tissues are embedded in a swellable hydrogel, to which the molecules and fluorescent tags in the samples are anchored. When the hydrogel swells several-fold, the effective resolution of the sample images can be improved accordingly via physical separation of the retained molecules and fluorescent tags. In this review, we focus on the early conception and development of ExM from a biochemical and materials perspective. We first examine the general workflow as well as the numerous variations of ExM developed to retain and visualize a broad range of biomolecules, such as proteins, nucleic acids, and membranous structures. We then describe a number of inherent challenges facing ExM, including those associated with expansion isotropy and labeling density, as well as the ongoing effort to address these limitations. Finally, we discuss the prospect and possibility of pushing the resolution and accuracy of ExM to the single-molecule scale and beyond.

## Introduction

Expansion microscopy (ExM) is an emerging bioimaging method that enables nanoscale fluorescence imaging of biological ultrastructure and molecular constituent via physical expansion of preserved cells and tissues. In a typical ExM workflow, biomolecules and fluorescent labels in the biological sample are first anchored to an expanding matrix, such as a swellable hydrogel. The hydrogel-embedded sample is then digested/homogenized to the molecular level, allowing it to swell several-fold linearly in water. During this swelling process, the polymer chains of the hydrogel pull away the neighboring biomolecules and fluorescent labels so that they can be differentiated on a diffraction-limited fluorescence microscope.

In this article, we set to review the recent progress in ExM that has enabled its application to nanoscale fluorescence imaging of intact cells and tissues. We first describe the working principle of a few existing super-resolution microscopy techniques and their significance to modern biomedical research (Sect. 2). We then introduce the basic concept and general workflow of ExM (Sect. 3). To date, a great number of bioconjugation and labeling strategies have been introduced to ExM in order to retain and visualize proteins, nucleic acids, membranes, glycans, saccharides, and small-molecules. We summarize the chemistries and biochemical processes behind these methods (Sect. 4). Lastly, we describe the recent progress in the field to address the inherent limitations of ExM, such as the challenges surrounding expansion isotropy and labeling density, as an on-going effort to further increase the technique’s resolution and accuracy (Sect. 5). Combined, our review provides a window into the early method development of ExM and its application to nanoscale fluorescence imaging.

## Nanoscale fluorescence imaging of biological samples

Light microscopy, which takes advantage of the optical contrast of biological samples, is one of the most widely used imaging techniques in modern biology and medical sciences. In comparison to electron microscopy and scanning probe microscopy, light microscopy allows molecule-specific labeling and three-dimensional (3D) visualization of biological structures in a minimally invasive manner. However, identifying cellular and subcellular components below the so-called diffraction limit, which is at the scale of hundreds of nanometers, has been a challenging task for light microscopy, because light, as a wave, is subject to diffraction at the far-field [1]. Hence, near-field optical microscopy, which detects non-propagating light waves from the sample surface with a nanoscale tip, has long been regarded as the only pathway to sub-diffraction-limit imaging [2].

The recent breakthrough in super-resolution microscopy has overcome this diffraction barrier at the far-field [3, 4]. Over the past few decades, a series of fluorescence microscopy methods have been developed to image biological structures beyond the hundreds-of-nanometer diffraction-limit. These include (and are not limited to) stimulated emission depletion (STED) microscopy, structured illumination microscopy (SIM), single-molecule localization microscopy (SMLM), pixel reassignment, and fluctuation-based super-resolution microscopy. In this section, we will review the working principle of some of these super-resolution microscopy techniques that have enabled nanoscale fluorescence imaging of biological samples.

### Stimulated emission depletion (STED) microscopy

**Fig. 1:**
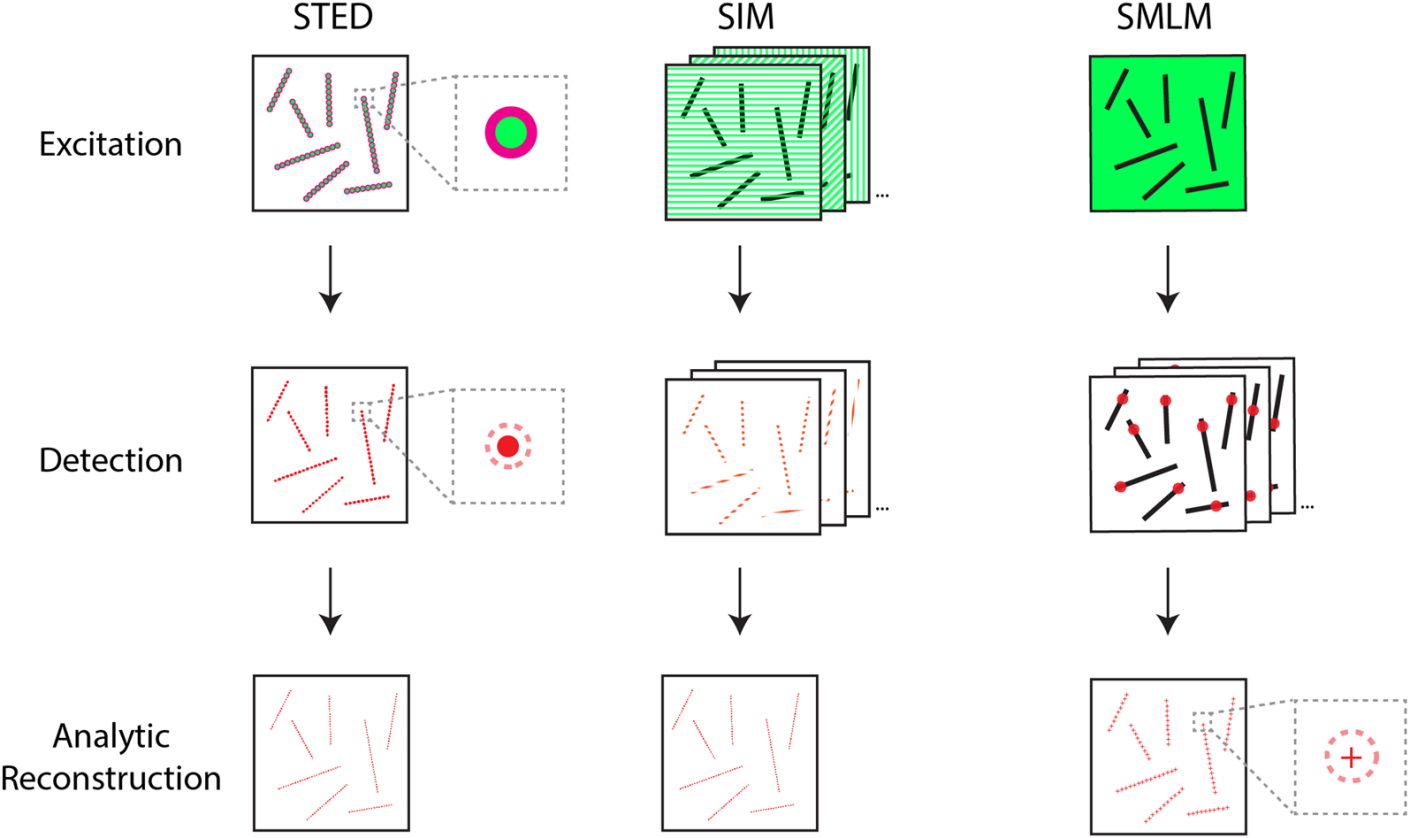
Imaging principle of super-resolution microscopy techniques such as stimulated emission depletion microscopy (STED), structured illumination microscopy (SIM), and single-molecule localization microscopy (SMLM). STED: During excitation, two laser beams—a laser beam producing a fluorescent spot with a diffraction-limited size (green) and a second laser beam depleting a donut-shaped area around the excited spot (magenta)—generate a sub-diffraction-limit fluorescent point for detection (solid red). The excitation/depletion spot is then scanned across the sample to generate the final image. SIM: Patterned excitations are first performed with multiple shifts and rotations. The partial fluorescent images from these excitation patterns are then detected and combined using an algorithm to reconstruct the final image of the sample. SMLM: In one implementation, a subset of photoswitchable fluorophores will turn on and off each time the sample is excited. During this process, only a sparse subset of the fluorophores—those at the on-state—are detected. After repeating the excitation-detection cycle many times, an increasing number of the single fluorescence points are captured. The centers of these single fluorescence points are then computationally determined and combined to reconstruct the final image of the sample

STED microscopy takes advantage of the nonlinear effect of two laser beams—one excitation beam with a conventional diffraction-limited spot and one depletion beam with a donut-shaped spot—to thin the excitation beam down to a sub-diffraction-limit spot [5, 6]. In more detail, the depletion beam (typically with a longer wavelength than the excitation beam) with the donut-shaped intensity profile will concentrically overlap with the excited area resulting from the excitation beam. The fluorophores in the overlapped area will be depleted before additional fluorescence events can take place (i.e., they will become non-fluorescent) (Fig. 1). As a result, a much smaller fluorescent spot, compared to the size of the diffraction limit, is produced. In practice, STED microscopy can achieve a resolution of ~ 40–110 nm in the lateral dimension and ~ 70–300 nm in the axial dimension [7]. In more recent years, femtosecond lasers and detector time gating are combined to improve the signal-to-noise ratio and reduce the background [8, 9]. In addition, substantial advancements have been made in multi-color STED imaging [10–12] and reducing photobleaching associated with the STED excitation [13, 14]. Finally, the concept of STED has been expanded to MINFLUX [15] and MINSTED [16, 17] nanoscopy to reach a spatial resolution of few nanometers and beyond.

### Structured illumination microscopy (SIM)

SIM takes advantage of high-spatial-frequency illumination patterns and reconstruction of the sample image excited by these patterns [18]. First, a series of images are obtained for the biological sample under the patterned illumination at different orientations. The most commonly used illumination patterns are patterned lines, but hexagonal or even random patterns can also be used [19]. The images from the high-frequency patterned excitations are then combined computationally to form the sample image, a process that makes use of Fourier transformation between the space and frequency domain [20]. In the case of patterned line illumination, multiple shifted and rotated excitation patterns are obtained using a grid or a spatial light modulator (Fig. 1) [21]. The final resolution of SIM can reach ~ 100–150 nm in the lateral dimension and ~ 250–350 nm in the axial dimension [7].

### Single-molecule localization microscopy (SMLM)

SMLM is a widely used super-resolution technique that takes advantage of the stochastic optical property of fluorescent proteins and organic dyes to single out the location of individual molecules [22, 23]. When the positions of individual fluorophores (i.e., fluorescent proteins and organic dyes) are recorded (using a diffraction-limited microscope), they will be fitted with a pre-determined intensity profile to precisely pinpoint their center positions. After repeating this process through multiple imaging rounds—collecting many location records of individual fluorophores—the individual pinpoints (i.e., centers of the fluorophores, typically at ~ 10–15 nm precision) obtained from these images are tabulated and combined computationally. Finally, a sub-diffraction-limit image consisting of numerous pinpoints is generated (Fig. 1).

The key in the SMLM process is to photoswitch (or photobleach or absorb/desorb) only a subset of the fluorophores so that the fluorescent ones can be distinguished from each other on a diffraction-limited optics system [24]. To date, a number of SMLM methods have been developed to achieve sparse activation/deactivation of photoactivatable and photoconvertible fluorophores, such as (fluorescence) photo-activated localization microscopy [(F)PALM] [25], (direct) stochastic optical reconstruction microscopy [(d)STORM] [26, 27], and to achieve stochastic absorption and desorption of fluorophores, such as points accumulation for imaging in nanoscale topography (PAINT) [28, 29]. Using SMLM, a spatial resolution of a few nanometers, namely ~ 5–30 nm in the lateral dimension and ~ 50–60 nm in the axial dimension, can be reached [7].

### Pixel reassignment and fluctuation-based super-resolution microscopy

A number of other microscopy techniques, such as pixel reassignment and fluctuation-based super-resolution microscopy, can also generate sub-diffraction-limit images at the far-field. Pixel reassignment super-resolution microscopy, for example, can resolve biological samples at ~ 120–150 nm resolution in the lateral dimension and ~ 350–450 nm resolution in the axial dimension [7, 30]. In this technique, an array detector rather than a single-point detector is used to capture the fluorescence signals obtained from the laser scan by using single or multiple focal spots. Then, the signals obtained from each element of the detector array are reassigned in space to achieve a smaller point-spread-function (PSF), resulting in higher resolution. In fluctuation-based super-resolution microscopy, the state transitions of the fluorophores (between the non-fluorescent and the fluorescent state) or the interactions between the fluorophores and the surrounding environment will randomly change the light emission of the fluorophores when excited with a continuous laser [31]. Using special algorithms, including that of super-resolution optical fluctuation imaging (SOFI) [32] and super-resolution radial fluctuations (SRRF) [33], the oscillations captured over a sequence of tens to hundreds of images can improve the imaging resolution to ~ 70–150 nm in the lateral dimension and ~ 450–600 nm in the axial dimension.

## Physical expansion of biological samples

Recently, a new nanoscale fluorescence microscopy technique called expansion microscopy (ExM), which requires no or minimal modifications to the optics system, was introduced [34]. The basic concept of ExM is to increase the size of the sample instead of improving the resolution of the fluorescence microscope itself [35, 36]. In a typical ExM workflow, the sample is embedded in a swellable hydrogel matrix, to which the biomolecules of interest in the sample are selectively anchored. After a mechanical homogenization step to break apart the sample at the molecular level, the sample-hydrogel composite is allowed to swell. When the hydrogel swelling occurs isotropically (i.e., when it expands uniformly in all directions), the relative spatial information of biomolecules at the pre-expansion state can be preserved. Finally, imaging of the physically enlarged sample can be performed using a (diffraction-limited) fluorescence microscope to achieve nanoscopic spatial resolution.

Nominally, ExM increases the effective spatial resolution by its linear expansion factor. The best lateral resolution of ExM reported to date is about tens of nanometers on a diffraction-limited microscope, which is comparable to that of existing super-resolution microscopy methods. Since ExM is a sample preparation technique, it is compatible with most of the fluorescence microscopy techniques, including the aforementioned super-resolution microscopy methods (see Sect. 5.2).

### Anchoring and in situ polymerization

**Fig. 2:**
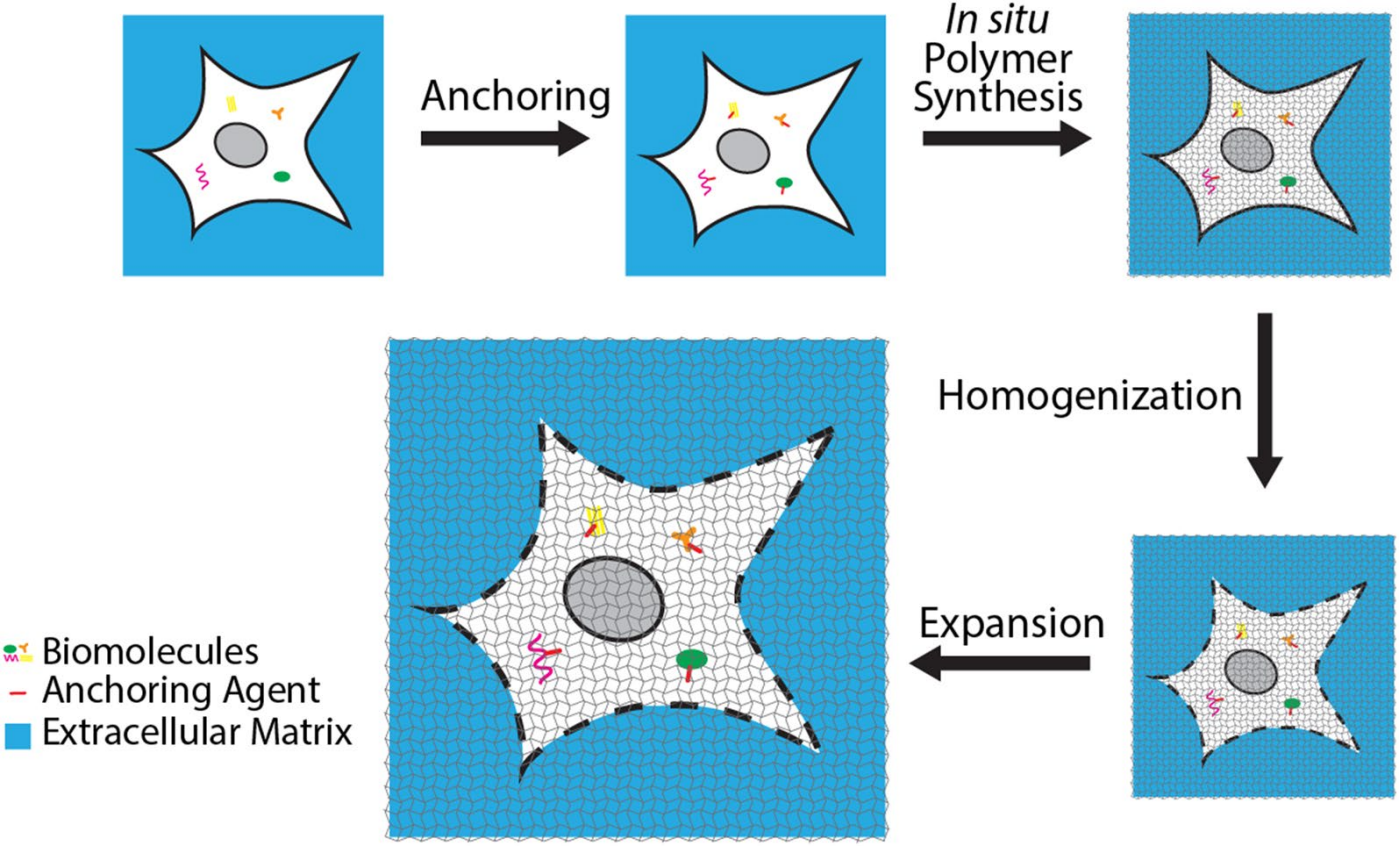
General workflow of expansion microscopy (ExM). Biological samples are first infused with a chemical anchor and polymerized in situ to form a sample-hydrogel composite. It then goes through a homogenization step, in which the molecules and structures that maintain the mechanical integrity of the sample are broken down. Finally, the sample-hydrogel composite is dialyzed in water for expansion

Most ExM variants reported to date follow a similar workflow, which consists of four major steps—anchoring, in situ polymerization, homogenization, and expansion (Fig. 2). First, biological samples are incubated with a chemical anchor such as acryloyl-X, SE (AcX) [37], methacrylic acid *N*-hydroxysuccinimide ester (MA-NHS) [38], and acrydite-modified oligonucleotides [34] (see Sect. 4.1). These chemical anchors are equipped with two or more functional groups, with one group to conjugate to the target biomolecules or fluorescent labels, and another to link to the hydrogel network. Next, the sample is incubated in a gelling solution, which is composed of the hydrogel monomers (e.g., acrylamide and sodium acrylate), cross-linking reagents (e.g., *N,N*′-methylenebisacrylamide), and if necessary, polymerization initiators [e.g., ammonium persulfate (APS) and *N,N,N*′*,N*′-tetramethylethylenediamine (TEMED)]. After the sample is infused with the gelling solution, it is allowed to go through a polymerization reaction in situ. In the case of the polyacrylamide/sodium polyacrylate gel, arguably the most commonly used hydrogels for ExM, the acrylamide and sodium acrylate monomers are cross-linked by *N,N*′-methylenebisacrylamide via free-radical chain-growth polymerization.

### Homogenization and expansion

After the sample is embedded in the hydrogel, the sample-hydrogel composite is homogenized to facilitate the subsequent expansion. The homogenization step plays an important role in ExM as it helps to break down the molecules and structures that maintain the structural integrity of the sample, such as lipids, structural proteins, and polysaccharides. It can be performed by treating the sample-hydrogel composite with heat and surfactant or by proteolysis (e.g., using enzymes like Proteinase K). After the homogenization step, the sample is softened and loosened, thereby becoming expandable along with the swelling hydrogel network. In addition, the homogenization step serves as an optical clearing step as it helps to remove optically absorbing and scattering structures and molecules, such as pigments and lipids. In the subsequent expansion step, these absorbing and scattering centers are washed away, resulting in a highly transparent sample-hydrogel composite with only the biomolecules of interest anchored to the polymer network of the gel.

Proteinase K is a commonly used enzyme for the homogenization step. It shows a strong enzymatic activity that breaks down almost any proteins into peptide fragments. One of the drawbacks of using Proteinase K digestion is, however, the potential loss of the fluorescent portions of the antibodies and fluorescent proteins (FPs) due to its strong digestive capability. For milder digestions, other enzymes can be used. Trypsin, for example, selectively cleaves the C-terminal of lysine or arginine residues, and thus can be used for a milder and more controllable homogenization process. However, additional validation of the expansion quality, including the evaluation of insufficient digestion and local tearing/breaking of the sample, is often needed for milder enzymatic digestions. Another milder homogenization method includes surfactant treatment [e.g., sodium dodecyl sulfate (SDS)] of the sample-hydrogel composite at high temperatures. This treatment serves to denature the proteins in the sample, and provides a much milder homogenization processcompared with the Proteinase K digestion.

Depending on the type and composition of the sample, additional homogenization agents and processes might be necessary. For example, when performing ExM on invertebrates, larvae, fungi, and plant cells that possess a rigid exoskeleton or cuticle made from lipids, polysaccharide, and glycoproteins, even the Proteinase K digestion may not be sufficient. In a recent report of expansion of *Drosophila* larvae, for example, an additional step of chitinase digestion is needed after the gelation and before the Proteinase K digestion to enable isotropic expansion [39]. To image the extracellular matrix of the *Drosophila* larvae, yet another digestion step with collagenase is required after the chitinase digestion and before the Proteinase K digestion [39].

Finally, expansion of the sample is achieved by dialysis of the sample-hydrogel composite (in water). Using the polyacrylamide/sodium polyacrylate gel as an example, sodium ions are diffused out of the hydrogel, while water is diffused into the hydrogel meshes during the dialysis. This results in a highly negatively charged environment within the hydrogel and leads to the swelling of the polyelectrolyte hydrogel network [35, 36].

### Sample staining

Depending on the goal of the experiments, sample staining can be performed before or after the expansion. These are so-called pre-expansion and post-expansion staining, respectively, with each having its own advantages and challenges. Pre-expansion staining labels the biological samples prior to the ExM process by introducing, for example, FPs or fluorescently-labeled antibodies to the sample. It is relatively straightforward to implement and is largely compatible with conventional genetic labeling and immunohistochemistry. While pre-expansion staining often provides adequate fluorescence signals after the ExM process, it can lead to degradation of the sample fluorescence. For example, organic dyes can be degraded or destroyed during the in situ polymerization step (e.g., degradation of the cyanine dyes by the free radicals) [40, 41]. In addition, as described earlier, the fluorescent portion of the antibodies or FPs can be removed during the homogenization step if not properly anchored to the hydrogel network. Finally, the pre-expansion staining approach often pre-determines the labeling density and precludes additional label amplification steps to be performed after the expansion. This imposes a limitation to the labeling density and signal-to-background ratio for the final images, as the density of the fluorescent labels is substantially diluted as the result of expansion.

In the post-expansion staining process, fluorescent labels are introduced after the gelation and homogenization step so that they are no longer degraded or destroyed during these processes. Furthermore, at the post-expansion state, more physical space is created around the target biomolecules so that additional labeling and amplification chemistries can be performed. The challenge of post-expansion staining, however, arises from the fact that the target molecules, including epitopes and other functional moieties to be recognized by the fluorescent labels, need to survive the polymerization and homogenization step. Often, additional optimization of the polymerization, homogenization, and labeling chemistries is required to establish a robust post-expansion staining workflow [42–44].

## Anchoring and visualization of biomolecules

The general workflow of ExM starts with (covalent) anchoring of the target biomolecules to the hydrogel network. The molecular information obtained from ExM will thus largely depend on this anchoring strategy. To date, a great number of molecular anchoring chemistries have been introduced to retain proteins, RNAs, lipids, and other chemical species to the ExM hydrogel. Table 1 summarizes a selected number of ExM methods that retain and visualize different types of biomolecules. In this section, we will review in more detail some of these anchoring strategies as well as the ExM variants that have adopted these strategies.

**Table 1:** Summary of ExM methods based on the target biomolecules

Target biomolecule	Method name	Chemical anchor(s)	Crosslinker(s)	Expansion factor (single round unless otherwise noted)	Commercial availability of the anchor(s)	References
Proteins	proExM	Acryloyl-X, SE	*N,N*′-Methylenebisacrylamide	~ 4–4.5×	Yes	[37]
	ExM compatible with conventional fluorescently labeled antibodies and fluorescent proteins	Methacrylic acid *N*-hydroxysuccinimide ester (MA-NHS), glutaldehyde (GA)	*N,N*′-Methylenebisacrylamide	~ 4×	Yes	[38]
	MAP	Acrylamide and formaldehyde	*N,N*′-Methylenebisacrylamide	~ 4×	Yes	[45]
	Original ExM/iExM	Acrydite-modified oligonucleotides	*N,N*′-Methylenebisacrylamide, *N,N*′-(1,2-dihydroxyethylene) bisacrylamide (DHEBA), *N,N*′*-*bis(acryloyl)cystamine (BAC)	~ 4.5–5.5×, ~ 16–22× (two rounds), ~ 53× (three rounds)	No	[34, 46]
	pan-ExM	Acrylamide and formaldehyde	*N,N*′-(1,2-dihydroxyethylene) bisacrylamide (DHEBA), *N,N*′-methylenebisacrylamide	~ 3.8–4×, ~ 13–21× (two rounds)	Yes	[47]
	TREx	Acryloyl-X, SE	*N,N*′-Methylenebisacrylamide	~ 10×	Yes	[48]
	X10 ExM	Acryloyl-X, SE	*N,N*-Dimethylacrylamide (DMAA)	~ 9.5×–11.5×	Yes	[49, 50]
	NIFS hydrogel	MA-NHS	*N,N*-Ethylenebisacrylamide (EBIS)	~ 9×	Yes	[51]
	ExR	Acryloyl-X, SE	*N,N*′-methylenebisacrylamide	~ 4×, ~ 20× (two rounds)	Yes	[52]
	Tetra-gel (TG)	NHS-azide	Step-growth polymerization (no crosslinking); the polymerization proceeds via click reaction between the azide and alkyne groups of the monomers	~ 3.0–3.5×, ~ 10–16× (two rounds), ~ 40× (three rounds)	Yes	[53, 54]
	LR-ExM	Trifunctional anchors (HOOC-MA-biotin, HOOC-MA-DIG, BG-MA-biotin, BG-MA-DIG, and BC-MA-DIG)	*N,N*′-Methylenebisacrylamide	~ 4.3–4.7×	No	[55]
	PhASE-ExM	Acryloyl-X, SE	8-arm and 10 kDa PEG-SH, *N,N*′-methylenebisacrylamide	~ 3.25×	Yes	[56]
	U-ExM	Acrylamide and formaldehyde	*N,N*′-Methylenebisacrylamide	~ 4×	Yes	[57, 58]
Nucleic acids	ExFISH	Label X	*N,N*′-Methylenebisacrylamide	~ 3.3×	No (Synthesized from Label-IT and AcX)	[59]
	EASI-FISH	MelphaX	*N,N*′-Methylenebisacrylamide	~ 2–3×	No (Synthesized from Melphalan and AcX)	[60]
	ExSeq	Label X	*N,N*′*-*Methylenebisacrylamide	~ 4×	No (Synthesized from Label-IT and AcX)	[61]
Lipids	mExM	Membrane probe pGk5b	*N,N*′-Methylenebisacrylamide	~ 3×	No	[62]
	LExM	Trifunctional LExM reagent	*N,N*′-Methylenebisacrylamide	~ 4–8×	No	[63]
	Sphingolipid ExM	α–NH_2_–ω–N_3_–C_6_–ceramide and GA	*N,N*′-Methylenebisacrylamide	~ 4 and 10×	No	[64]
Multiplexed labeling	TRITON	Trivalent TRITON linker	*N,N*′-Methylenebisacrylamide	~ 3.2×	No	[40, 65]
	Click-ExM	Interaction between biotin, streptavidin, and Acryloyl-X, SE (or GA)	*N,N*′-Methylenebisacrylamide	~ 4.5×	No	[66]
Physical entanglement	eMAP	Entanglement by the polymer chains (no covalent anchoring)	*N,N*′-Methylenebisacrylamide	~ 4 and 10×	Yes	[67]
	ELAST	Entanglement by the polymer chains (no covalent anchoring)	*N,N*′-Methylenebisacrylamide	~ 2–3×	Yes	[68]

### Protein retention

Proteins are an essential component of living organisms with diverse functions to maintain and regulate biological activities. The early development of ExM has been focused on retaining proteins in the preserved samples. In this type of ExM, proteins are commonly anchored to the hydrogel network using a small-molecule anchor, followed by the sample homogenization and expansion step. The physical distances between the anchored proteins are increased, resulting in a better resolved fluorescence image across the sample. Endogenously expressed fluorescent proteins (FPs) as well as conventional immunohistochemistry are generally compatible with the protein-retention version of ExM. In this section, we will review different anchoring strategies developed for protein retention in ExM.

#### Acryloyl-X, SE (AcX)

AcX is a bifunctional linker with a methacryloyl group capable of participating in the free-radical chain-growth polymerization and an NHS ester group capable of conjugating to the residual primary amines of proteins. When antibodies and endogenous (fluorescent) proteins are modified by AcX, they become anchorable to the ExM hydrogel network in the in situ polymerization step. This version of ExM, which is based on AcX-modification of proteins, is called protein-retention ExM (proExM) [37, 41]. In proExM, fluorescent reporters are often introduced pre-expansion as endogenously expressed proteins (e.g., FPs) and later introduced antibodies (e.g., dye-conjugated primary or secondary antibodies).

In another variant of proExM, unlabeled proteins in the sample can be fluorescently labeled post-expansion via immunostaining or other methods. The post-expansion staining proExM process provides several advantages compared to the pre-expansion staining proExM, as it can avoid the degradation of fluorescent proteins and organic dyes (e.g., cyanine dyes) during the polymerization and homogenization step. More importantly, the post-expansion staining allows a more uniform staining to be performed across thick tissue samples, because the sample-hydrogel composite stays microscopically porous after the expansion. However, additional optimization of the homogenization step is often needed to better preserve the epitopes.

**Fig. 3:**
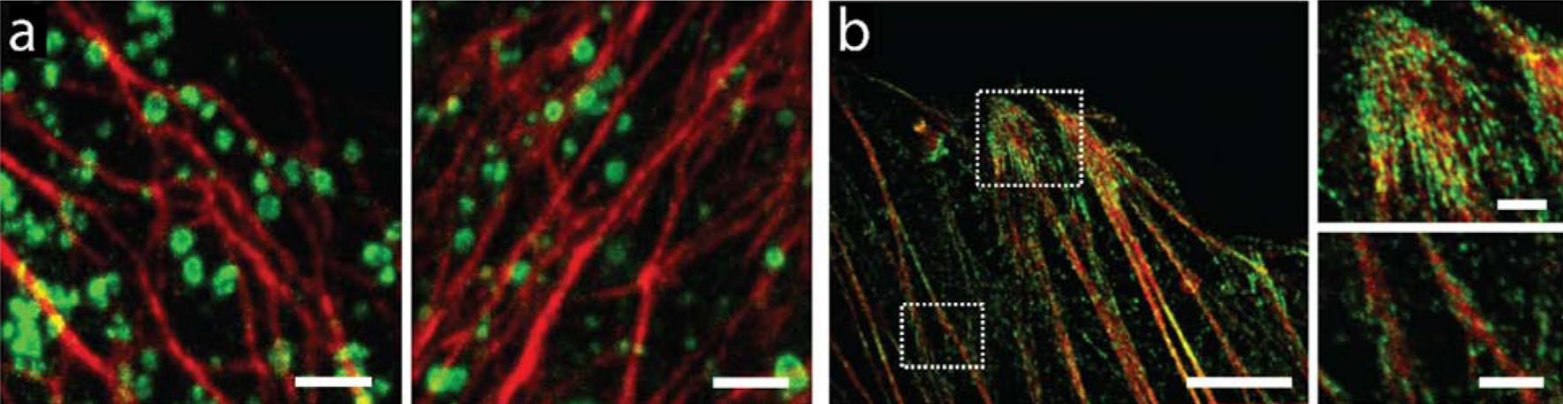
Protein-retention ExM (proExM) of HeLa cells using acryloyl-X, SE (AcX) as the protein anchor. **a** Dual-color proExM of clathrin (fused to mEmerald, green) and keratin (mRuby2, red). Two representative images are shown. Scale bar, 1 μm (4.3 μm; here and after, physical size after the expansion, if available, is shown in brackets). **b** Dual-color proExM image of actin (mRuby2, red) and paxillin (mEmerald, green) fusions. Insets are magnified views of the boxed regions. Scale bars, 5 μm (21.5 μm) and 1 μm (4.3 μm, insets). (Reprinted/adapted by permission from Spring Nature: Tillberg et al. [37], copyright 2016)

**Fig. 4:**
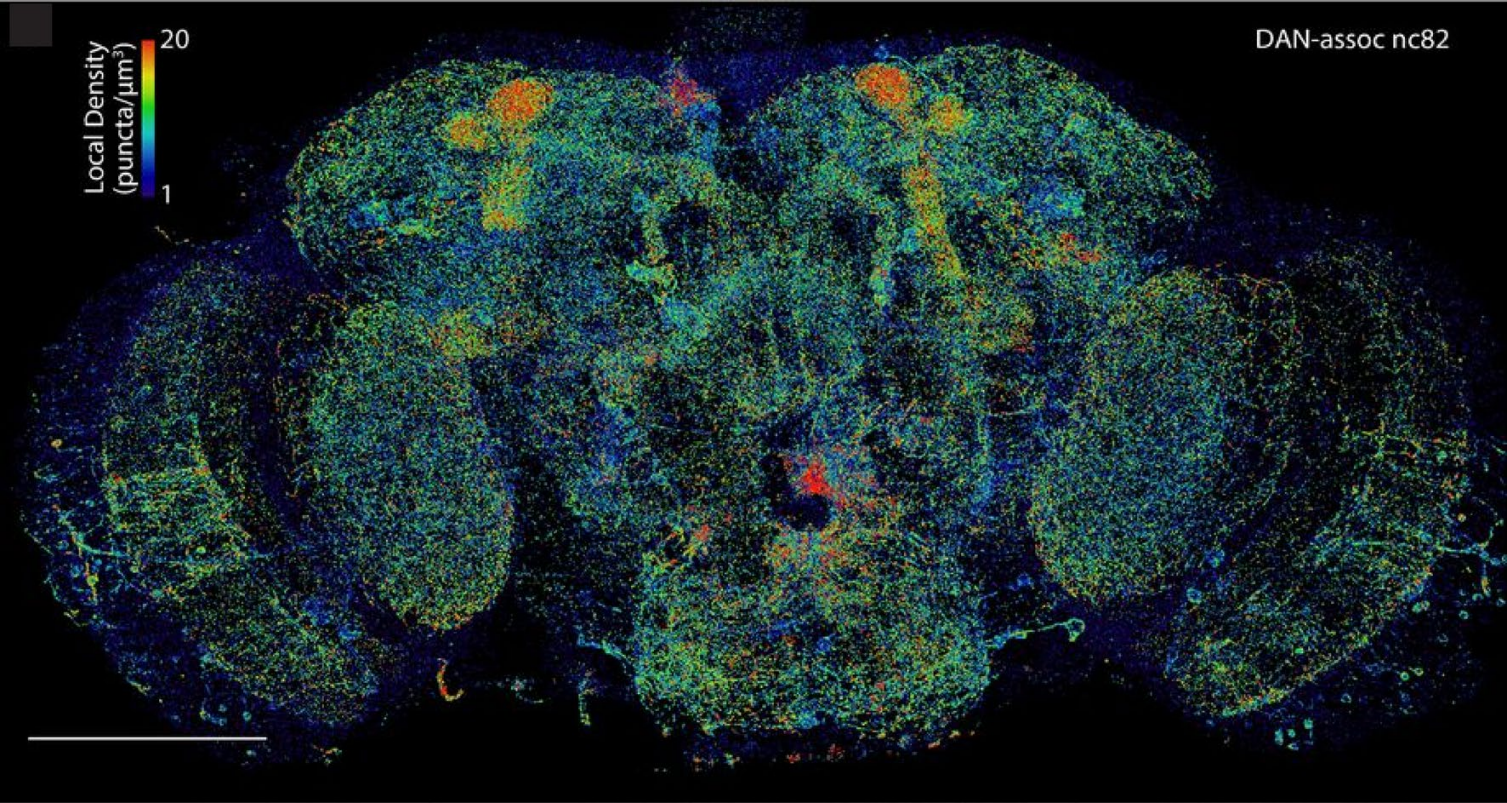
Combining proExM and lattice light sheet microscopy achieves whole-brain imaging of *Drosophila* with sub-100 nm resolution. Scale bar, 100 μm. (From Gao et al. [70]. Reprinted/adapted by permission from AAAS)

AcX is commercially available and can be easily accessed. In other words, no in-house design or synthesis of special reagents is required for proExM, making it arguably one of the most popular protein anchors. In practice, proExM is used to retain and visualize proteins in various types of samples, including *C. elegans* [69], *Drosophila* [39, 70, 71], zebrafish [72, 73], non-human primates [37], human clinical samples [74–76], organoids [56], and plants [77, 78]. Using proExM, for example, microtubules of HeLa cells have been imaged at a resolution of ~ 70 nm (Fig. 3) [37]. In another example, the entire brain of *Drosophila*, prepared by a modified version of proExM, has been imaged at sub-100 nm resolution using lattice light-sheet microscopy (Fig. 4) [70]. Yet in a different example, Photo-transfer by Allyl Sulfide Exchange Expansion Microscopy (PhASE-ExM) enables ~ 3.25× physical expansion and super-resolution visualization (~ 124 nm) of proteins in organoids with AcX-based protein tethering to the hydrogel network [56].

#### Methacrylic acid *N*-hydroxysuccinimide ester (MA-NHS)

MA-NHS, another commercially available chemical anchor, has a molecular structure similar to AcX but with a smaller molecular weight. The working principle of MA-NHS is also similar to that of AcX: its NHS ester group reacts with the residual primary amines of proteins, and its methacryloyl group participates in the free-radical chain-growth polymerization. As a result, endogenous proteins and antibodies in the biological samples can be anchored to the ExM hydrogel network after the MA-NHS modification.

**Fig. 5:**
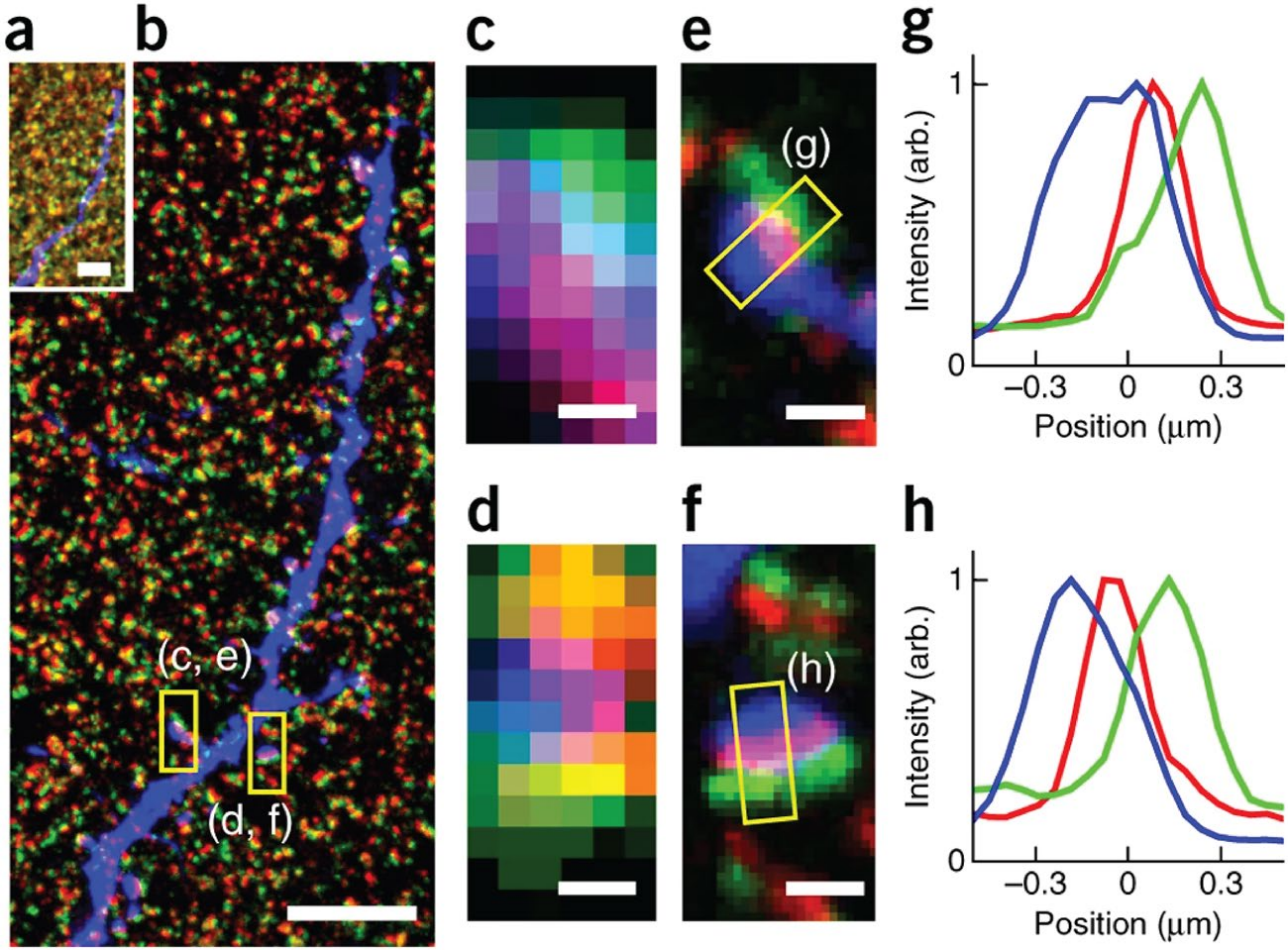
Expansion of Thy1-YFP-H mouse brain slice using MA-NHS as the protein anchor. **a** Triple-color proExM of endogenously expressed YFP (blue), immunostained presynaptic marker Bassoon (green), and immunostained postsynaptic marker Homer1 (red). Scale bar, 5 μm. **b** The same area as in **a** after expansion. Scale bar, 5 μm. **c**, **d** Magnified views of the boxed regions before expansion. Scale bars, 500 nm. **e**, **f** The same area in **c** and **d** after expansion. Scale bars, 500 nm. **g**, **h** Cross-sectional profiles of the boxed regions in **e** and **f** (arb., arbitrary units). (Reprinted/adapted by permission from Springer Nature: Chozinski et al. [38], copyright 2016)

ExM based on MA-NHS modification is compatible with conventional immunohistochemistry and can be applied to a wide range of samples. For example, presynaptic (Bassoon) and postsynaptic markers (Homer1) in an immunostained Thy1-YFP-H mouse brain slices have been visualized at sub-100 nm resolution using the MA-NHS anchoring strategy (Fig. 5) [38].

#### Glutaraldehyde (GA)

**Fig. 6:**
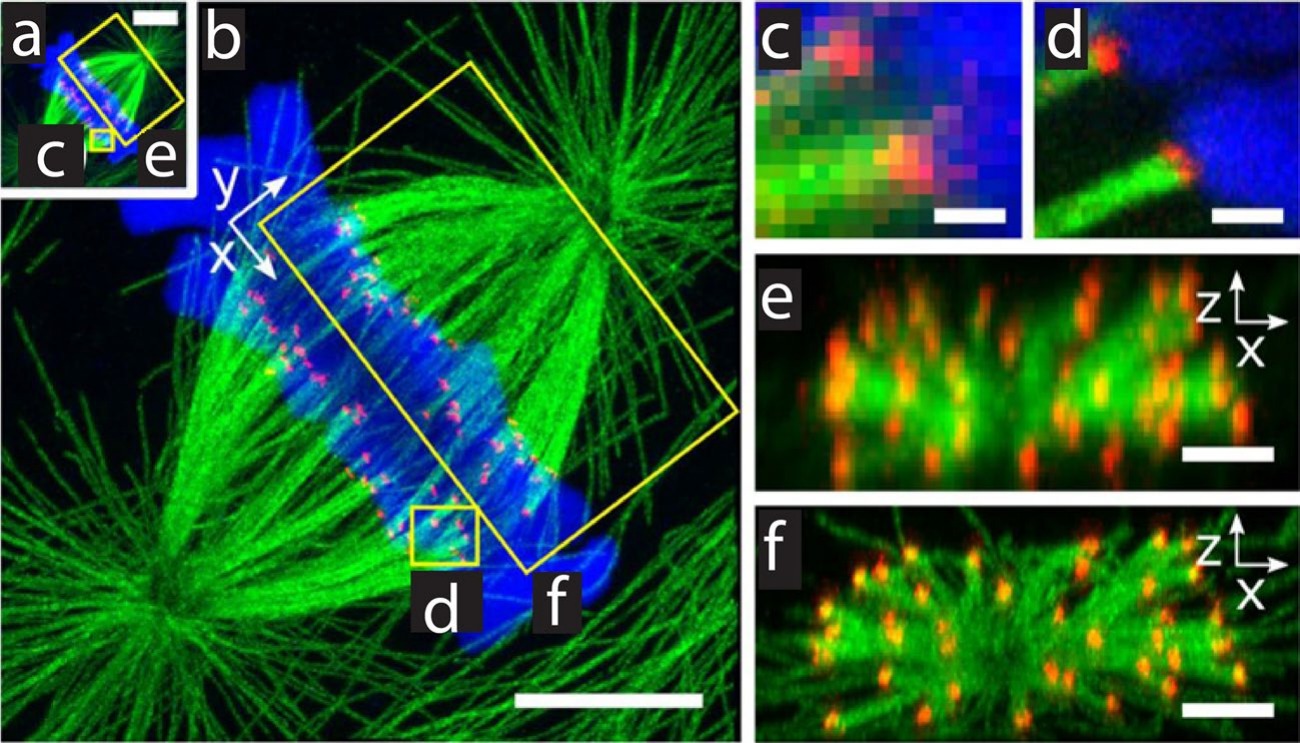
Pre- (**a**) and post-expansion (**b**) images of a dividing PtK1 cell immunostained for tubulin (green) and the kinetochore protein HEC1 (red). Scale bar, 5 μm. **c**, **d** Magnified views of microtubule-kinetochore attachments from the boxed regions in **a** and **b**. Scale bars, 500 nm. **e**, **f** End-on views of the boxed regions in **a** and **b**. Scale bars, 2 μm. (Reprinted/adapted by permission from Springer Nature: Chozinski et al. [38], copyright 2016)

GA in aqueous solution establishes an equilibrium between the monomeric form and the polymeric form. The polymeric form of GA is equipped with both an aldehyde functional group, which can conjugate to the N-terminal of proteins, and an alkene group, which can be anchored to the ExM hydrogel network. GA is more commonly used for cell expansion with pre-expansion staining. This is because GA may introduce higher fluorescence background in the tissue and need additional processing for post-fixation immunostaining. GA-based fixation and anchoring of proteins and the subsequent expansion has been demonstrated with, for example, immunostained PtK1 cells (Fig. 6) [38], yeast [79], and fungi [80].

#### Formaldehyde and acrylamide

By mixing in a high concentration of acrylamide to formaldehyde, the intra- and interprotein crosslinking effect of the formaldehyde solution, which is commonly used for sample fixation, can be suppressed. This allows the acrylamide to directly react with the methylol groups that are formed from the residual amines of proteins reacting with formaldehyde. As a result, the reactive bridge formed across the acrylamide, formaldehyde, and amine can be tethered into the polyacrylamide/sodium polyacrylate hydrogel network. This acrylamide/formaldehyde anchoring strategy was previously used in polymer-based tissue clearing and expansion methods, such as CLARITY and PACT [81–87]. The same strategy has been demonstrated for expansion and post-expansion immunostaining to realize protein retention in the expanded samples in the recently reported magnified analysis of the proteome (MAP) [45] and ultrastructure expansion microscopy (U-ExM) [57]. With MAP, for example, multiscale proteomic imaging of intact biological samples, as large as a whole organ, is possible. When combined with advanced molecular preservation methods, MAP further allows the extraction of molecular identity, subcellular architecture, and intercellular connectivity of diverse cell types at sub-diffraction-limited resolution [88].

#### Acrydite-modified oligonucleotides

**Fig. 7:**
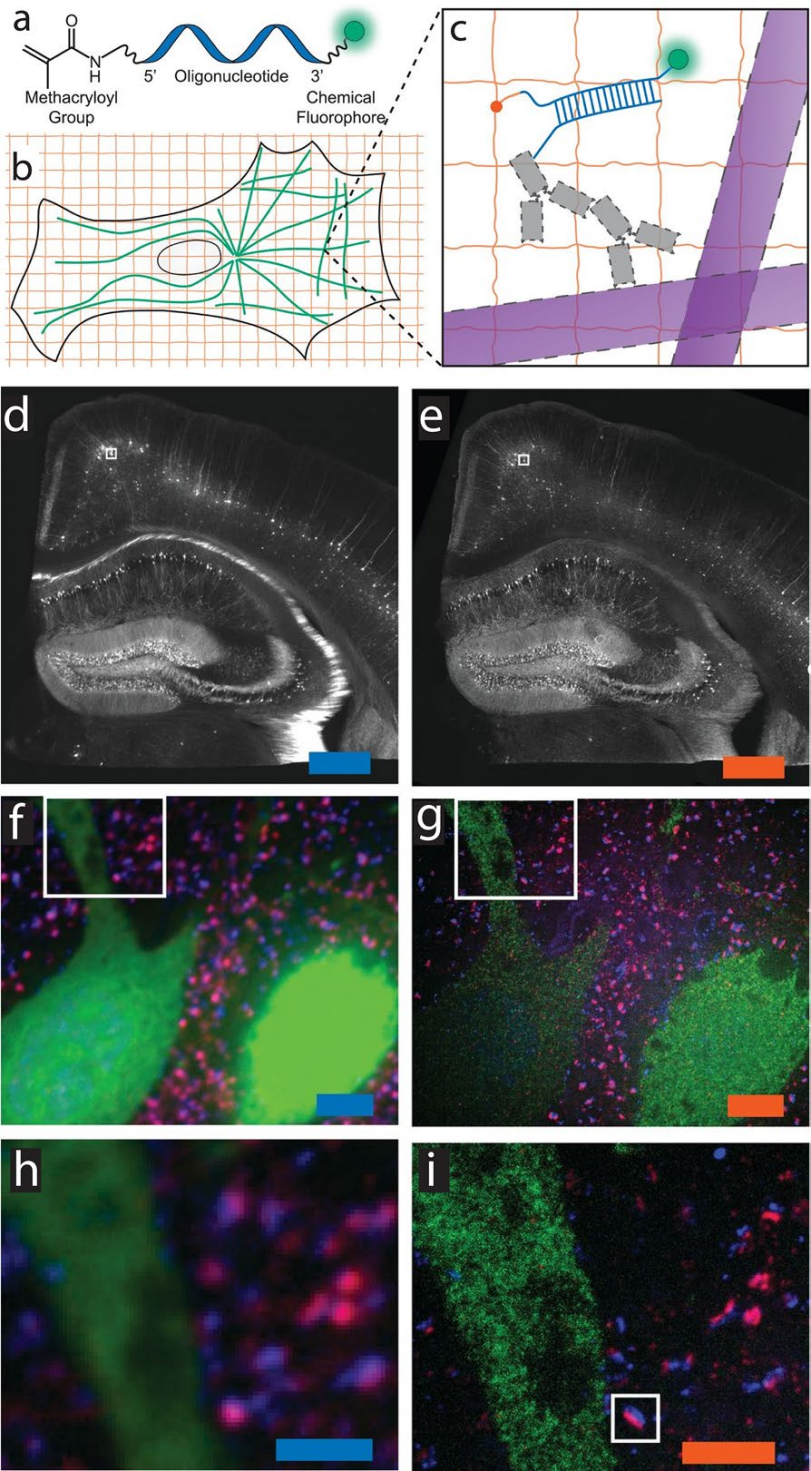
Anchoring and labeling of proteins using acrydite-modified oligonucleotides (oligos) in the original ExM protocol. **a** Chemical structure of the acrydite-modified oligo. **b** Schematic of microtubules (green) and hydrogel network (orange). **c** The acrydite-modified oligo (shown in **a**) hybridized to a oligo-bearing secondary antibody (gray, top), bound via the primary antibody (gray, bottom) to the microtubules (purple). The acrydite-oligo is incorporated into the gel (orange lines) via the methacryloyl group (orange dot) and remains anchored after the removal of  the microtubules and antibodies by proteolysis (dotted lines). **d** Pre-expansion widefield fluorescence image of Thy1-YFP mouse brain slice. Scale bar, 500 μm. **e** Post-expansion widefield image of the same sample in **d**. Scale bar, 500 μm (2.01 mm). **f**, **g** Confocal fluorescence image of the boxed regions in **d** and **e**, respectively. Scale bars, 5 μm (20.1 μm) **h**, **i** Confocal fluorescence image of the boxed regions in **f** and **g**, respectively. Scale bars, 2.5 μm (10.0 μm). (From Chen et al. [34]. Reprinted/adapted by permission from AAAS)

In the original version of ExM, an acrydite-modified, dye-conjugated oligonucleotide (oligo) is used as an anchor (as well as a fluorescent reporter) to retain the spatial information of the target proteins (Fig. 7) [34]. Through the specific hybridization between the acrydite-modified oligo and the complementary oligo conjugated to an antibody, the target protein can be specifically anchored to the swelling hydrogel network. However, the protein itself does not necessarily need to stay anchored, because the acrydite-modified oligo itself retains the protein’s spatial information. In this case, the acrydite-modified oligo serves more like an anchorable fluorescent label rather than the anchor of the proteins. This oligo-based anchoring/labeling strategy has been adopted by the original ExM as well as the iterative expansion microscopy (iExM) protocol [46].

### Nucleic acid retention

Spatial analysis of transcriptomic, genomic, and epigenomic information has revolutionized our understanding of gene expression and regulation in living organisms. Similar to protein-retention, preservation of nucleic acids can be achieved by using a small-molecule anchor that directly links these molecules to the ExM hydrogel network. For post-expansion visualization, one of the commonly used techniques is fluorescence in situ hybridization (FISH). FISH probes are functionalized oligos with complementary sequences to the target RNAs or DNA. They are commonly equipped with a fluorescence reporter (or a moiety to develop fluorescence through additional chemistries) for the subsequent fluorescent readout. The location of the target RNAs or DNA sequences can then be determined by the location of the fluorescence reporters.

#### Alkylation of nucleic acids

**Fig. 8:**
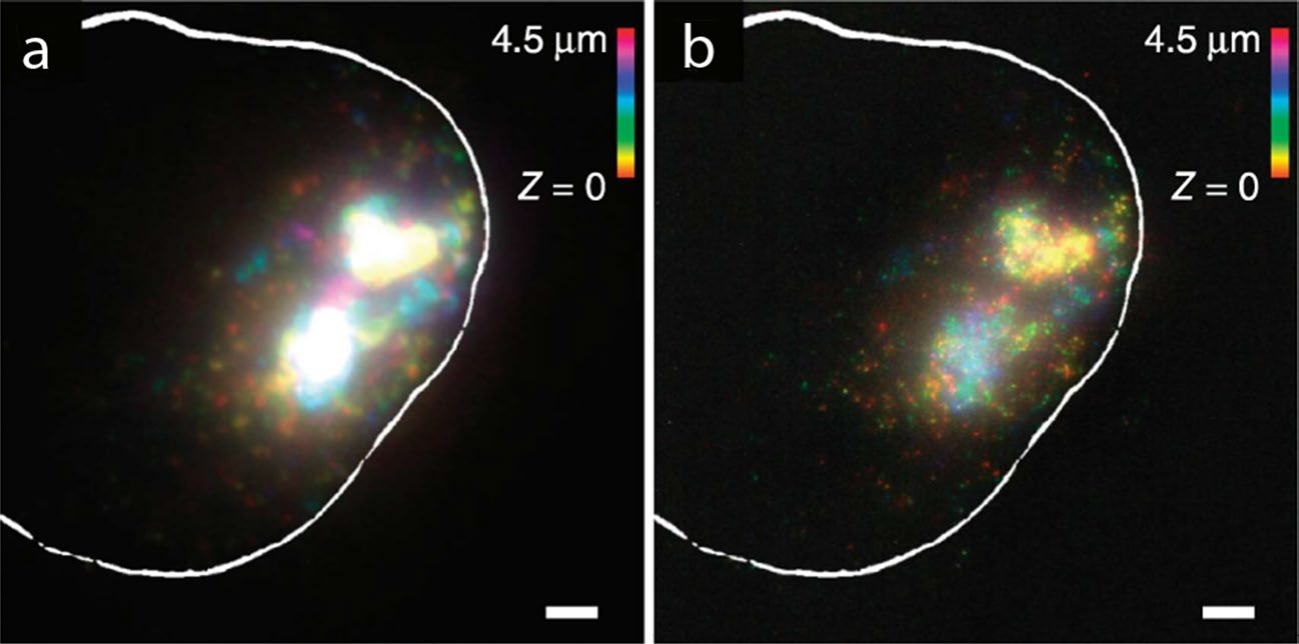
**a** Single-molecule FISH (smFISH) image of XIST long non-coding RNA (lncRNA) in the nucleus of an HEK293 cell before expansion (the white line denotes the nuclear envelope). **b** As in **a**, using Expansion Fluorescence In Situ Hybridization (ExFISH). Scale bars, 2 μm (6.6 μm). (Reprinted/adapted by permission from Springer Nature: Chen et al. [59], copyright 2016)

One popular ExM variant that retains and visualizes RNAs is called Expansion Fluorescence In Situ Hybridization (ExFISH) (Fig. 8) [59]. In the typical ExFISH workflow, a small-molecule chemical anchor is first infused throughout the sample to covalently link the RNAs to the hydrogel network. To date, several small-molecule anchors have been validated for ExFISH. LabelX is prepared by mixing Label-IT amine with AcX. In this synthesis, the NHS ester group of AcX reacts with the terminal amine group of Label-IT amine. As a result, LabelX retains the reactive group from Label-IT that can selectively alkylate guanine, one of the nucleotides in nucleic acids. The methacryloyl group of AcX (now part of LabelX) can then anchor the nucleic acids to the polymer network by participating in the free-radical chain-growth polymerization.

MelphaX is another small-molecule anchor for nucleic acids, which has been used in the recently developed EASI-FISH protocol [60]. MelphaX is synthesized by mixing Melphalan and AcX, and has two alkylation moieties for RNA anchoring. Compared to LabelX, MelphaX has been validated to increase the brightness of individual RNA spots and to improve the signal-to-noise ratio by 25%.

Alkylation of nucleic acids using LabelX or MelphaX is compatible with the aforementioned AcX-based protein retention. By combining these two anchoring strategies, proteins and RNAs (and DNA) can be concurrently retained and visualized in the same ExM sample [59]. In ExFISH and EASI-FISH, close to 2–3× expansion can be obtained as the FISH probes need to be imaged in a diluted buffer in which the polyelectrolyte ExM hydrogel shrinks slightly. Importantly, ExFISH is compatible with various signal amplification and multi-round imaging strategies. This includes scalable labeling strategies such as MERFISH [85, 89]. More recently, an iterative multi-round expansion process called Expansion Revealing (ExR) has achieved retention of RNAs and the subsequent FISH labeling with close to 20× expansion [52].

**Fig. 9:**
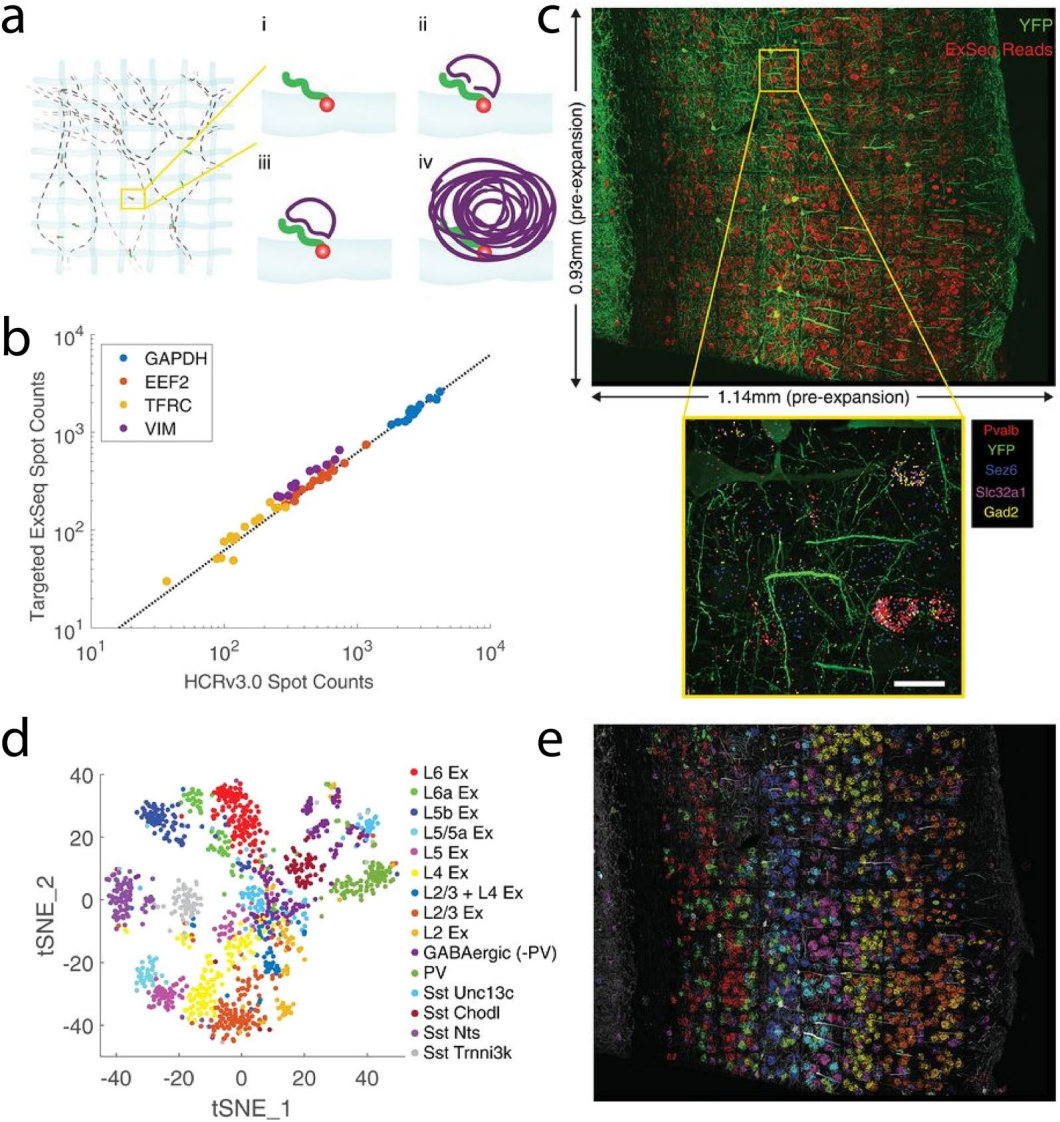
Spatial mapping of RNAs using Expansion Sequencing (ExSeq). **a** Targeted ExSeq library preparation: (i) RNA anchoring and expansion, (ii) padlock probe hybridization, (iii) probe ligation, and (iv) rolling circle amplification. **b** Amplicon counts for targeted ExSeq versus HCRv3.0-amplified ExFISH for the same transcript in the same HeLa cell (60 cells). **c** Targeted ExSeq of 42 cell type marker genes in Thy1-YFP mouse visual cortex. (Top) Fluorescence image showing targeted ExSeq reads (red) and YFP (green). (Bottom) Localization of marker genes Pvalb (red), Sez6 (cyan), Slc32a1 (magenta), and Gad2 (yellow) with YFP (green). Scale bar, 20 μm. **d** Targeted ExSeq gene expression profiles of 1154 cells clustered into 15 cell types. **e** Spatial organization of cell types identified in **d**. Cell-segmented reads are shown, colored by cluster assignment, and overlaid on YFP (white). (From Alon et al. [61]. Reprinted/adapted by permission from AAAS)

Expansion Sequencing (ExSeq) combines LabelX-based RNA anchoring and fluorescence in situ and/or ex situ sequencing (Fig. 9) [61]. On top of the hybridization-based targeted detection of the anchored RNAs, ExSeq can also provide the sequence information in an untargeted manner, which utilizes in situ spatial barcoding and ex situ sequence matching. Beyond spatial transcriptomics, ExSeq can be applied to in situ mapping of cellular lineage and/or connectome-indexing RNA barcodes with randomized base-level variant that is not easily resolved by conventional FISH-based readout. In addition, ExSeq is compatible with multiplexed fluorescence imaging of oligo-barcoded antibodies.

#### Trifunctional anchors

**Fig. 10:**
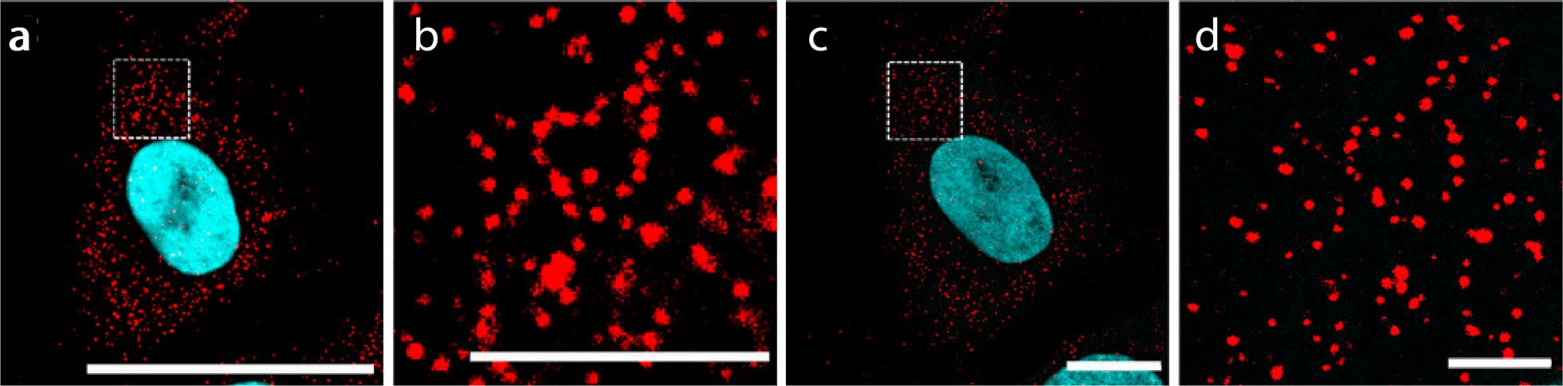
RNA detection by combining ExM and hybridization chain reaction (HCR) v3.0 using a TRITON linker. **a** Pre-expansion image of PGK1 transcripts. **b** Magnified view of the boxed region in **a**. **c** Post-expansion image of the same cell. **d** Magnified view of the boxed region in **c**. Scale bars, 50 μm (**a**, **c**) and 10 μm (**b**, **d**). (Reprinted/adapted with the permission from Wen et al. [40]. Copyright 2021 American Chemical Society)

Nucleic acids can be anchored to the hydrogel network via custom-synthesized trifunctional anchors, such as TRITON (also see Sect. 4.4.2) [40]. TRITON is equipped with a hybridization chain reaction initiator for signal amplification, a readout probe which targets the RNA of interest, and a polymerizable acryloyl moiety that helps tether the target RNA to the hydrogel. Since the reactive group for the RNA hybridization is designed to target specific RNA species, TRITON is often used for targeted ExFISH (Fig. 10).

#### Indirect anchoring through histone proteins or by entanglement

Indirect anchoring of genomic DNA via retention of the histone proteins has been used for DNA FISH-based detection of genomic regions, such as in expansion pathology (ExPath) [74], U-ExM [58], and TRanslational Epigenetic Encoding (SCEPTRE) [90]. Furthermore, physical entanglement of the genomic DNA by the hydrogel polymer network has also been explored for non-covalent anchoring. For example, the chromatin structure in intact mouse embryos has been visualized by combining direct hydrogel embedding and in situ and/or ex situ sequencing in the recently reported in situ genome sequencing (IGS) [91].

### Membrane retention

Biological membranes are essential for compartmentalization of (sub)cellular structures. They also play an important role in numerous cellular signaling pathways [92–95]. Due to the complexity of lipid chemistry, however, it is challenging to molecularly label the biological membrane, and to image its ultrastructure using light microscopy. Retention of the spatial information of cellular membranes has therefore become one of the focuses of methodology development in ExM.

#### Intercalating molecules

In membrane ExM (mExM), an exogenous amphiphilic probe with a sidechain of lysine is first infused throughout the sample [62]. This probe, which readily intercalates the lipid membrane structure, is composed of a lipid tail on the N-terminal of the lysine chain, with a glycine in between to provide mechanical flexibility. d-Lysine, rather than the biological active l-lysine, is used as the backbone to minimize its degradation during the homogenization step of Proteinase K digestion. The C-terminal of the lysine chain is conjugated to a biotin that can react with one of the four binding sites of fluorescently labeled streptavidin. The remaining active sites of streptavidin can be further reacted with biotinylated fluorophores to amplify the fluorescence signal. Finally, once infused into the membrane structures, the probe can be anchored to the hydrogel matrix via the residual primary amines on the lysine chain using AcX or MA-NHS.

**Fig. 11:**
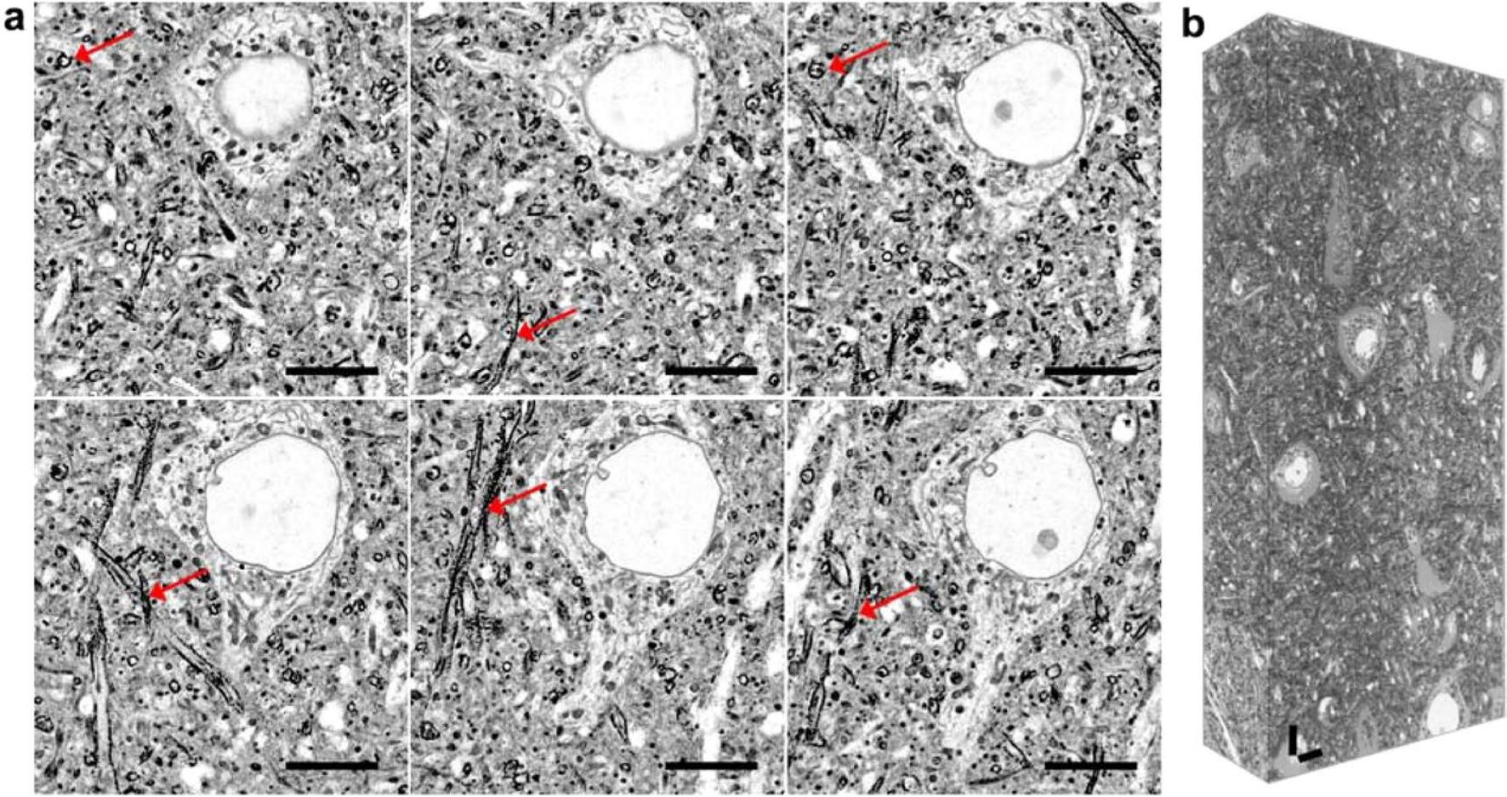
Membrane expansion microscopy (mExM) of fixed brain tissue. **a** Six serial sections from a 3D image stack obtained on a confocal spinning-disk microscope. Axons can be identified by their high contrast due to the increased concentration of lipids in the myelin sheaths (red arrows). Scale bar, 10 μm. **b** mExM processed tissue imaged with light-sheet microscopy. Scale bar, 10 μm. (Reprinted/adapted from Karagiannis et al. [62] by permission from the author)

mExM has been used to image, for example, membrane structures in a fixed mouse brain tissue using confocal and light-sheet fluorescence microscopy (Fig. 11) [62]. Additionally, the mExM anchoring strategy is compatible with concurrent lipid and protein retention and visualization. For example, it has been used to co-visualize the membranes as well as immunostained structures in the mouse brain tissue, which include the endoplasmic reticulum surface protein (Calnexin), Golgi apparatus marker (Giantin), mitochondrial membrane protein (Tom20), a nuclear pore complex component (NUP98), and myelin sheaths (Myelin Basic Protein, MBP) [62].

#### Metabolic labeling

Another strategy for membrane retention is metabolic labeling. In a recent report, a modified sphingolipid is used to image the nanoscopic changes of membrane structures during bacterial infection (“sphingolipid ExM”) [64]. In sphingolipid ExM, a custom-synthesized sphingolipid (a short-chain ω-N3-C6-ceramide bearing a clickable azide group) is metabolically incorporated into the plasma membrane and the inner membranes of HeLa cells. After fixation, permeabilization, and reaction with DBCO-AF488 (via click chemistry), the cellular membrane structures containing the synthesized sphingolipids are fluorescently labeled. Finally, ~ 4–10× expansion with an effective resolution of ~ 10–70 nm can be achieved after dialyzing the sample-hydrogel composite in water. To date, sphingolipid ExM has been applied to study the sphingolipid structure in cellular membranes and to visualize intracellular pathogens and their interactions with mitochondrial proteins after the cells are fed with pathogens. Click-ExM is another strategy to retain and visualize the membrane structures via metabolic labeling and click chemistry [66] (see Sect. 4.4). The recently developed Lipid Expansion Microscopy (LExM) also combines metabolic labelling and click chemistry to achieve nanoscale imaging of the plasma and organelle membrane [63].

### Multifunctional and universal anchors

Apart from the biomolecule anchoring strategies described so far, ExM imaging of other molecular species, such as saccharides, glycans, and small molecules, is substantially more challenging, due to the limited bioconjugation and labeling chemistries developed to date. To overcome this limitation, multifunctional anchor, which allows the fluorescence reporter to be linked to the anchor rather than the biomolecules themselves, has been developed for ExM. Click chemistry-based anchoring (also known as Click-ExM) and TRITON are two recent examples of introducing multifunctional anchors to the ExM process. With these new developments, more than one types of biomolecules can be anchored to the hydrogel network and fluorescently labelled. This anchoring/labeling strategy opens up the possibility of multiplexed study of molecular functions and interactions in the same ExM sample.

#### Click chemistry-based anchoring

Click chemistry is a biorthogonal chemistry with specific reactivity between two functional groups. Click chemistry can be implemented by, for example, copper-catalyzed azide-alkyne cycloaddition (CuAAC) or copper-free, strain-promoted azide-alkyne cycloaddition (SPAAC). Taking advantage of click chemistry-based bioconjugation, Click-ExM combines multiplexed molecular anchoring and ExM to enable super-resolution imaging of multiple biomolecules in the same sample [66]. Click-ExM can be used to retain and visualize proteins, lipids, nucleic acids, glycans, saccharides, and certain small molecules.

In Click-ExM, synthetic biomolecules with azide functional groups are either metabolically introduced or passively infused throughout the sample. The azide groups are then reacted with an alkyne-biotin via click chemistry, followed by a staining step using fluorescently labeled streptavidin. Finally, the streptavidin is anchored to the hydrogel matrix via AcX, and the remaining procedure follows the general ExM workflow. In the Click-ExM process, the synthetic molecules with the azide groups thus play a dual role of an anchor and a fluorescent reporter.

**Fig. 12:**
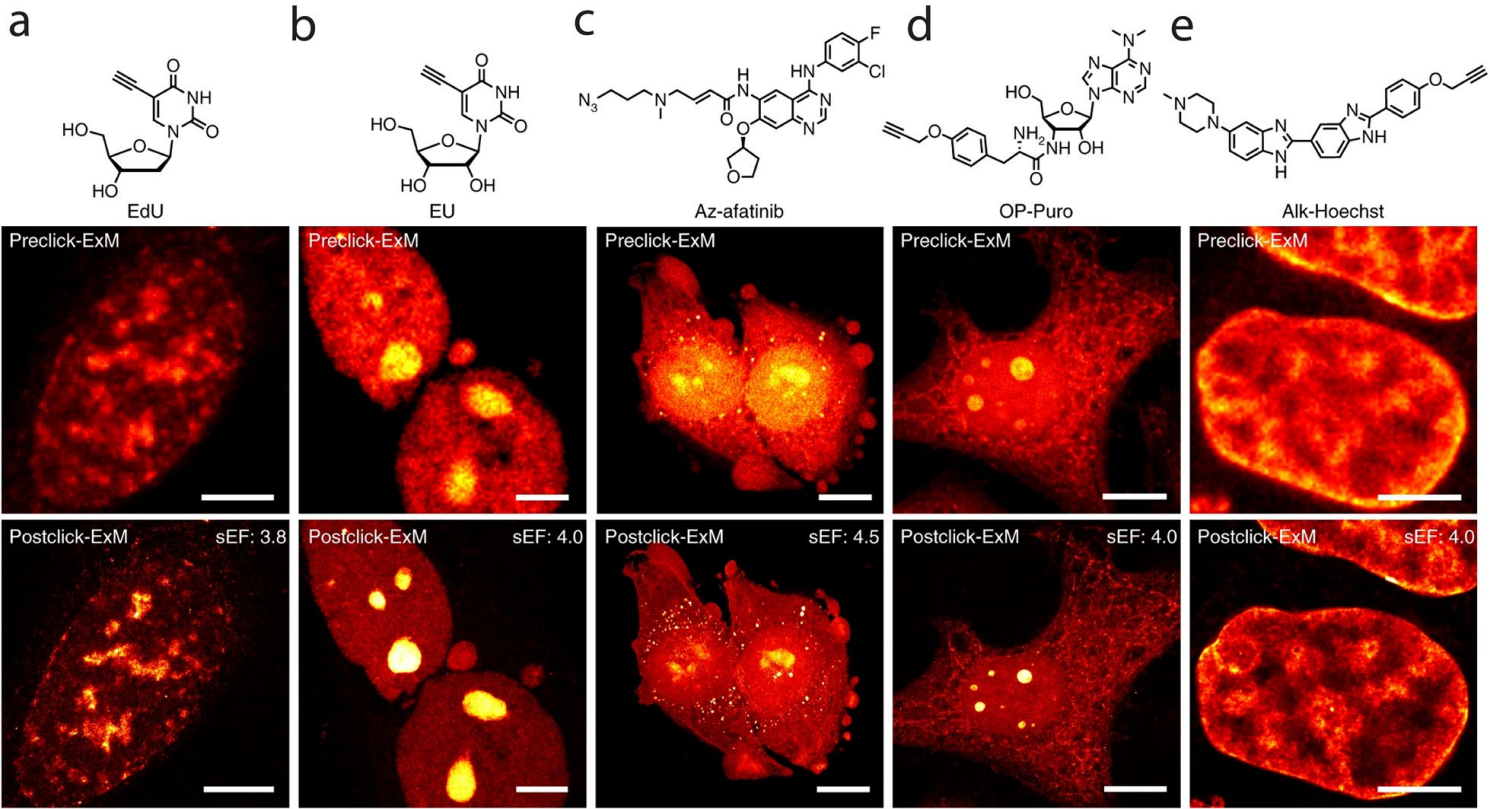
Click-ExM imaging of proteins, nucleic acids and small molecules. **a** Chemical structure of EdU, and pre- and post-click-ExM images of nascent DNA (AF488) in EdU-treated HeLa cells. Scale bar, 5 μm. **b** Chemical structure of EU, and pre- and post-click-ExM images of nascent RNA (AF555) in EU-treated HeLa cells. Scale bar, 5 μm. **c** Chemical structure of Az-afatinib, and pre- and post-click-ExM images of HeLa cells treated with Az-afatinib (AF555). Scale bars, 10 μm. **d** Chemical structure of OP-Puro, and pre- and postc-lick-ExM images of nascent peptides (AF488) in OP-Puro-treated HeLa cells. Scale bar, 10 μm. **e** Chemical structure of Alk-Hoechst, and pre- and post-click-ExM images of DNA (AF488) in Alk-Hoechst-labeled HeLa cells. AcX was used for anchoring. Scale bar, 5 μm. All the scale bars are in the pre-expansion dimension. (Reprinted/adapted by permission from Springer Nature: Sun et al. [66], copyright 2021)

Different Click-ExM variants have been developed to retain and visualize various molecular species, including proteins, nucleic acids, small molecules, and glycans (Fig. 12). For example, Click-ExM has enabled retention and nanoscale imaging of glycans by introducing an azide-modified sialoglycan to the sample.

#### Trivalent anchoring (TRITON)

**Fig. 13:**
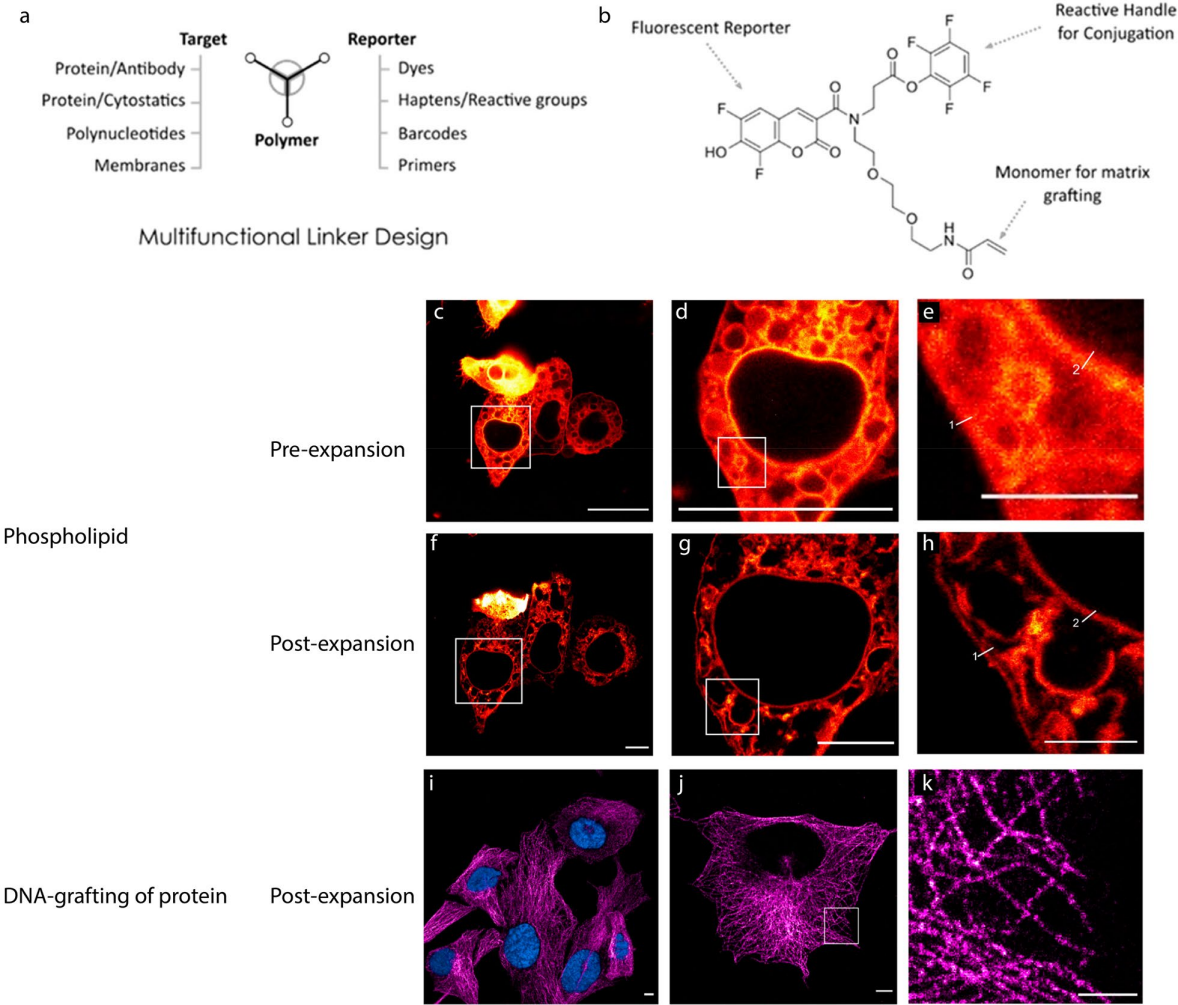
ExM using trivalent anchoring (TRITON). Molecular design (**a**) and example (**b**) of a TRITON trivalent linker with a fluorescent reporter (Pacific Blue), reactive tetrafluorophenyl (TFP) ester for amine conjugation, and acrylamide monomer for grafting to the expansion microscopy polymer. **c**–**h** Expansion of HeLaP4 phospholipid membranes through lipid conjugation to a fluorescent TRITON trivalent linker. **c**–**e** Pre-expansion image (**c**) with magnified views (**d**, **e**) of the boxed areas. Scale bars: 25 μm (**c**, **d**) and 5 μm (**e**). **f**–**h** Post-expansion image (**e**) with magnified views (**g**, **h**) of the boxed areas. Scale bars: 7.8 μm (25 μm) (**f**, **g**) and 3.03 μm (10 μm) (**h**). **i**–**k** Immunostaining of α-tubulin of HeLa cell via direct grafting of oligo-conjugated secondary antibody and fluorescent oligo-based readout post-expansion. **i** Two-color imaging with a nuclear DAPI staining (cyan) and immunostaining of α-tubulin, visualized with readout oligo (Cy5,magenta). **j** The same staining as **i** but with the DAPI-channel removed to show the high signal-to-noise ratio and low background staining in the nucleus. **k** Magnified view of the boxed area in **j**. Scale bars: 3.03 μm (10 μm) (**i**, **j**) and 1.52 μm (5 μm) (**k**). (Reprinted/adapted with the permission from Wen et al. [65]. Copyright 2020 American Chemical Society)

Another effort of developing a universal anchor for ExM has led to the design and synthesis of a trifunctional linker called TRITON [40, 65]. TRITON is composed of three functional moieties covalently linked to one another, including a reactive group that targets different biomolecules, a reporter group that reports the location of the targeted biomolecules, and a grafting moiety that participates in the free-radical chain-growth polymerization. In TRITON-based ExM, the fluorescent reporters are directly linked to the anchor. Therefore, loss of fluorescence can be reduced during the homogenization step (as the anchor will not be digested during this step) (Fig. 13) [40, 65].

### Anchoring through entanglement

So far, almost all the anchoring strategies take advantage of the covalent bonds formed between the target molecules or fluorescent labels and the hydrogel network. Instead of using a covalent anchor, physical retention of the endogenous biomolecules has also been explored for ExM. In epitope-preserving magnified analysis of proteome (eMAP), for example, physical entanglement with the hydrogel network serves to retain the endogenous biomolecules and their nanoscopic organizations at ~ 4–10× expansion [57, 67]. Since chemical modifications to the biomolecules are minimized in this protocol, eMAP offers improved preservation of epitopes. Using eMAP, multiplexed, multi-round nanoscopic characterization of individual synapses has been demonstrated for both ~ 4× and ~ 10× expanded tissue.

The same entanglement strategy is used in entangled link-augmented stretchable tissue-hydrogel (ELAST) [68], a recently developed tissue processing method to render the brain tissue stretchable and compressible. ELAST can be used, for example, to rapidly and reliably immunostain thick brain tissues, while elastically and reversibly changing the tissue thicknesses prior to the immunostaining. For example, millimeter-scale slabs from the human and non-human primate brains have been immunostained using ELAST.

## Resolution and expansion isotropy

The ability of ExM to retain and visualize a variety of biomolecules with nanoscale resolution has drastically expanded its applicability and practicality (see Sect. 4). To date, a great number of anchoring and labeling strategies have been developed and optimized for ExM. With these new chemistries, ExM can now be applied to high spatial-resolution mapping of proteins, RNAs, DNA, lipids, glycans, saccharides, and small-molecules in intact cells and tissues.

Another important aspect of the ongoing method development for ExM focuses on enhancing the method’s resolution and imaging quality. This involves (a) increasing the expansion factor, (b) increasing the sample labeling density, and (c) reducing the local distortion associated with the expansion. In this section, we will review the new chemistries and implementations of ExM behind these efforts.

### Higher expansion factor

Expansion factor, the ratio of the post-expansion to the pre-expansion sample size, determines the effective resolution of ExM. In principle, the larger the expansion factor is, the better the resolution ExM can achieve (with the caveat that the expansion isotropy can affect the imaging resolution). To date, high-expansion-factor ExM has been demonstrated by using single-round expansion with a highly swellable hydrogel or by consecutively performing the expansion process multiple times.

#### Single-round expansion

Altering the chemical composition of the ExM hydrogel drastically changes its swelling capability. For polyelectrolyte hydrogels (e.g., the polyacrylamide/sodium polyacrylate gel), two major factors influence the final expansion factor. One factor is the hydrophilic ionic sidechains. When placed in water, the charges on the ionic sidechains of the hydrogel electrostatically repel each other and act to swell the hydrogel network. As a general rule, the more ionic sidechains the polyelectrolyte hydrogel has, the larger expansion factor it can achieve after dialysis. The other factor is the concentration of the crosslinkers. Less cross-linking commonly leads to an increase of the expansion factor(while also resulting in reduced mechanical stability of the gel).

**Fig. 14:**
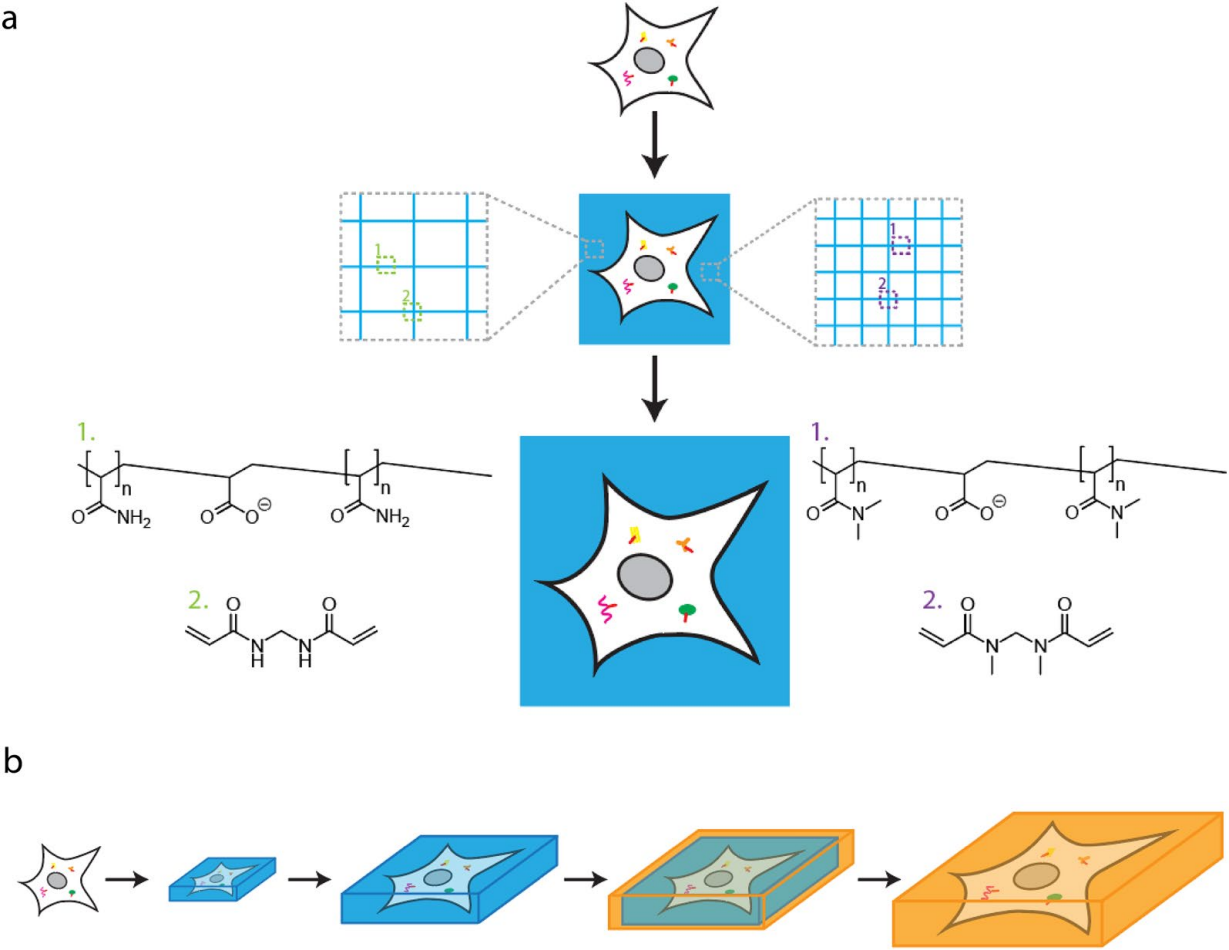
ExM variants with higher expansion factors. **a** Single-round expansion with high expansion factor (~ 10×). The chemical composition of the hydrogel is schematically illustrated for Ten-fold Robust Expansion Microscopy (TREx, left) and X10 Expansion Microscopy (right). **b** Iterative expansion. First, the sample is embedded in an expanding hydrogel (blue). Next, the expanded sample-hydrogel composite is re-embedded in a second expanding hydrogel (orange) and expanded once again

To further increase the expansion factor of ExM, these two factors can be tuned from the original ExM hydrogel recipe (which achieves ~ 4–4.5× expansion) (Fig. 14a). For example, a single-round ~ 10× expansion can be achieved by systemically changing the composition of the polyacrylamide/sodium polyacrylate gel (Ten-fold Robust Expansion Microscopy or TREx) [48]. This includes modifying the concentration of the crosslinker (*N,N*′-methylenebisacrylamide), the concentration of the monomers (acrylamide and sodium acrylate), and the gelation temperature from the original ExM hydrogel recipe [34]. In this optimization process, the mechanical properties of the hydrogels (synthesized using various compositions) have also been taken into account so that the optimized gels can be handled easily. Notably, the exact expansion factor of the TREx gel, which can reach as large as ~ 10×, can vary according to the samples and the gelation environments (e.g., the shapes of the gelation chamber). Therefore, one may want to optimize the gelation condition for each specimen type and gelation setup [48].

Instead of modulating the monomer and the cross-linker concentrations, a hydrogel with a stronger swelling property can be used to achieve higher expansion factor. In X10 Expansion Microscopy, for example, superabsorbent *N,N*-dimethylacrylamide (DMAA) and sodium acrylate copolymers are used to achieve up to ~ 10× expansion [49, 50]. In this gel (“DMAA gel”), DMAA forms the main polymer chains and the sparse self-crosslinkers while sodium acrylate serves to boost the swelling by providing the ionic sidechains. Similar to the proExM protocol, AcX can be used to anchor proteins to the DMAA gel.

**Fig. 15:**
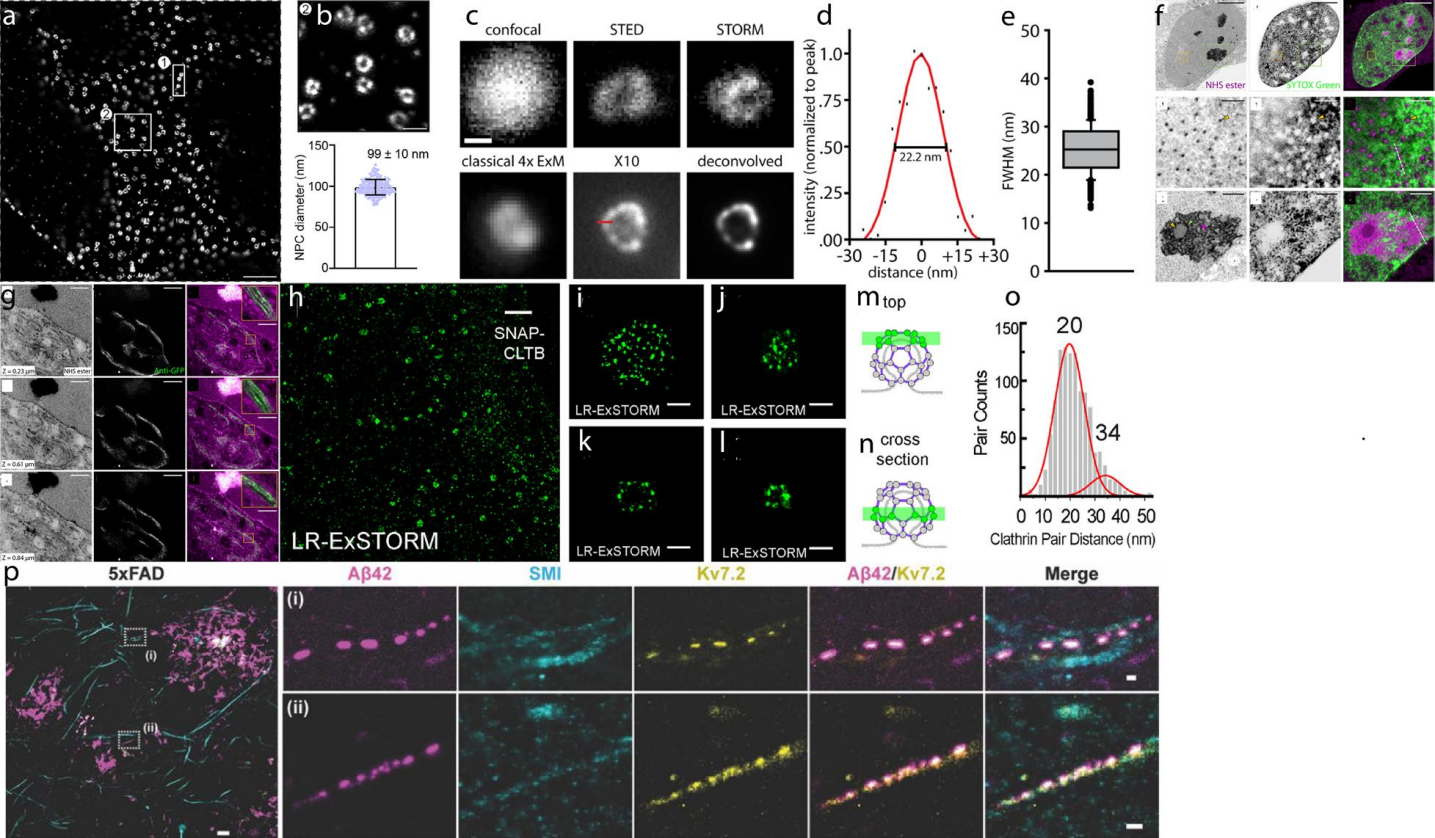
High-resolution images obtained with high-expansion-factor ExM variants. **a** Nucleus imaged by confocal microscopy after TREx. Scale bar, 1 μm. **b** High-resolution view of several nuclear pores from boxed region (2) of **a** and the distribution of diameters of individual nuclear pores. Scale bar, 200 nm. **c** Immunostainings for the peroxisome membrane protein Pmp70 in neurons by both super-resolution and X10 Expansion Microscopy. Scale bar, 100 nm. **d** The exemplary line scan from the X10 Expansion Microscopy image in **c** is shown with a best Gaussian fit curve. **e** Quantification of the average resolution of X10 Expansion Microscopy image, which is 25.2 ± 0.2 nm. **f** pan-ExM reveals nuclear architecture in interphase. Image of SYTOX Green nucleic acid stain (middle column) and NHS ester pan-stained sample (left column) and overlay of them (right column). Scale bars, (top row) 5 μm, (middle row) 250 nm, (bottom row), 1 μm. **g** Overlay of the NHS ester and anti-GFP images. The insets show the zoom-in yellow boxes and reveal individual ManII-positive Golgi cisternae. Scale bars, 2 μm. **h** Label-retention expansion STORM (LR-ExSTORM) image of a HeLa cell overexpressing SNAP-CLTB, stained with BG-MA-biotin, and post expansion labeled with streptavidin-AF647. Scale bar, 2 μm. **i**, **j** Images of x–y cross sections at the top of single CCPs as illustrated in **m**. Scale bar, 100 nm. **k**, **l** Images of x–y cross sections in the middle of single CCPs as illustrated in **n**. Images in **i**–**l** are different CCPs. Scale bar, 100 nm. **o** Nearest cluster distance analysis of 134 CCPs imaged with LR-STORM. **p** Expansion Revealing (ExR) reveals co-localized clusters of Aβ42 peptide and potassium ion channels in the fornix of Alzheimer’s model 5× FAD mice. The image showing post-expansion Aβ42 (magenta), SMI (cyan) and Kv7.2 (yellow) staining in the fornix of a 5xFAD mouse (Scale bar, 400 nm). The left most panel, merged low magnification image (Scale bar, 4 μm). (**a**, **b** Reprinted/adapted from Damstra et al. [48]. **c–e** Reprinted/adapted from Truckenbrodt et al. [49]. **f**, **g** Reprinted/adapted from M’Saad et al. [47]. **h**–**o** Reprinted/adapted by permission from Rockefeller University Press: ©2021 Shi et al. [55]. Originally published in *J. Cell. Biol.* 10.1083/jcb.202105067. **p** Reprinted/adapted from Sarkar et al. [52] by permission from the author)

Both TREx and X10 Expansion Microscopy have been validated to achieve a higher expansion factor compared to the ~ 4–4.5× expansion in the original ExM. For example, resolution tests using nuclear pore complexes (NPCs) with NUP96-GFP tag have validated the expansion factor of TREx to be ~ 9.5× (Fig. 15) [49]. Combining BODIPY-FL NHS staining (a universal protein labeling) and TREx, subcellular structures, such as the neuropil region outside the cell, nucleus of each cell, the nuclear envelope, and organelles in cytosol can all be visualized. Finally, expansion of peroxisomes, ~ 100–200 nm in size, has verified the effective resolution of X10 Expansion Microscopy to be ~ 25 nm (Fig. 15).

In addition, a single-round ~ 9× expansion has been reported recently [51]. In this method, instead of AcX and *N,N*′-methylenebisacrylamide, methacrylic acid *N*-hydroxysuccinimide ester (MA-NHS) and *N,N*-ethylenebisacrylamide (EBIS) are used as the protein anchor and hydrogel crosslinker, respectively. The polyacrylamide/sodium polyacrylate gel with 0.06% (w/w) EBIS concentration has achieved the highest expansion factor (~ 8.92×), which corresponds to an effective resolution of 31.5 ± 4.3 nm. The final ninefold swelling (NIFS) hydrogel enables ~ 30 nm resolution imaging on a conventional fluorescence microscope by single-round expansion of ninefold.

#### Iterative expansion

The iterative expansion process takes advantage of concatenation of one hydrogel network by another, and the sequential swelling provided by each hydrogel (Fig. 14b). In a typical workflow of iterative expansion, the sample is first expanded by a swellable hydrogel and then re-embedded and re-expanded in a second swellable hydrogel. If the expansion factor of both gels is 4.5×, the final expansion factor can reach as high as 20× (= 4.5 × 4.5). The re-embedding/re-expansion process can be repeated to achieve an even higher expansion factor.

In iterative Expansion Microscopy (iExM), standard polyacrylamide/sodium polyacrylate gel with ~ 4–4.5× expansion factor is used to implement the sequential expansion [46]. For the first round of expansion, *N,N*′-(1,2-dihydroxyethylene) bisacrylamide (DHEBA) is used as the crosslinker, so that it can be chemically cleaved after being re-embedded in another gel. Furthermore, in iExM, the locations of the target proteins are retained using acrydite-modified oligos (see Sect. 4.1), so that the spatial information can be transferred across different gels by using gel-anchorable and complementary oligos. In a typical workflow, the target protein is first labeled with an oligo-conjugated antibody. The oligo is then hybridized with a complementary acrydite-modified oligo, which is subsequently anchored to a cleavable DHEBA gel (the first-round expanding gel). After homogenization and expansion, the first-round expanding gel is re-embedded in an intermediate, cleavable, and non-expanding gel (also formed using DEHBA as the crosslinker, but without sodium acrylate) in order to maintain the first gel at its expanded state. The oligo specifying the location of the proteins is then hybridized with another acrydite-modified oligo with the complementary sequence, before the sample-hydrogel composite is re-embedded in a second-round expanding hydrogel with conventional non-cleavable crosslinker. After cleaving both the first-round expanding gel and the intermediate non-expanding gel, the sample-hydrogel composite is expanded the second time to reach the final expansion factor.

Other ExM methods that take advantage of the multi-round, iterative expansion include pan-ExM [47, 96] and expansion revealing (ExR) [52]. In pan-ExM, proteins are first anchored to a polyacrylamide/sodium polyacrylate gel using the acrylamide/formaldehyde fixation method (see Sect. 4.1). After the first round of expansion and re-embedding in another non-expanding hydrogel, the acrylamide/formaldehyde fixation method is performed once again to anchor the previously masked primary amines of proteins to the second-round expanding hydrogel. Finally, the first-round expanding gel and the non-expanding re-embedding gel are chemically cleaved, before the second-round expansion takes place (Fig. 14). The final expansion factor of pan-ExM is ~ 13–21× after two rounds of expansion [47].

Expansion revealing (ExR) also uses two-round iterative expansion to achieve close to 20× expansion (Fig. 15) [52]. In ExR, instead of cleaving the first-round gel and the re-embedding gel, they are kept chemically intact. In other words, the second-round expansion takes place while stretching the polymer chains of the first-round (and the reembedding) gels. One advantage of this process is that by removing the chemical cleaving step and by retaining the first-round expanding gel, biomolecules covalently anchored to the first gel can be retained after the second round of expansion. This eliminates the need for labeling transfer across the gels, rendering the iterative expansion process compatible with direct anchoring of biomolecules to the first-round gel (e.g., AcX anchoring of proteins). The inter-layer distances of synaptic proteins, such as PSD95, Homer1, and Shank3, have been used to validate the resolution of ExR to be ~ 20 nm (Fig. 15) [52].

Tetra-gel (TG) is a recently reported hydrogel formed by click chemistry-based step-growth polymerization of two complementary, tetrahedral monomers with sodium polyacrylate and PEG backbones [54]. As described later (see Sect. 5.4), TG is designed for high-isotropy expansion with sub-10 nm spatial errors. This new hydrogel is compatible with the iterative expansion process, and can be used as the first-round expanding gel. The cleavable TG is formed by introducing a cleavable SS-DBCO end-functional group to one of the monomers. After expanding and re-embedding, the cleavable TG’s polymer network can be decomposed at the monomer level for the second-round expansion. Using TG as the first-round expanding gel, up to three rounds of iterative expansion of intact virus particles has been demonstrated [54].

### Combining ExM and super-resolution microscopy

ExM is a sample preparation technique that enables sub-diffraction-limit fluourescence imaging on a diffraction-limited microscope. Apart from increasing its expansion factor, the final resolution of ExM can also be improved by combining it with existing super-resolution microscopy methods. In practice, a few issues need to be addressed before such imaging can happen. For example, some super-resolution microscopy methods require a special buffer as the imaging medium, which may decrease the expansion factor of gel. Another potential issue is the expansion itself, which may cause the sample to be out of the working distance of objectives conventionally used for super-resolution microscopy. Finally, the labeling in the sample is diluted when the size of the sample increases. This may lead to lowered signal-to-background ratio, faster photobleaching, and/or punctation of the imaged structures in the final images. In this section, we will review a few strategies and methods to overcome these challenges.

#### Expansion STED (ExSTED)

**Fig. 16:**
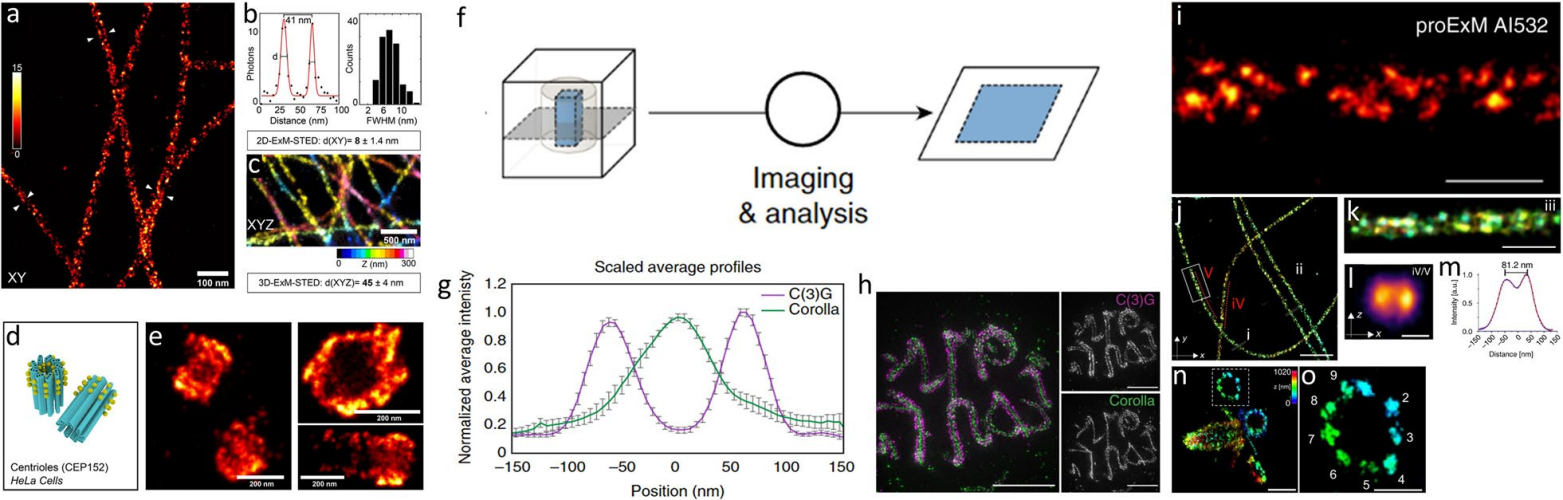
Combining ExM and super-resolution microscopy methods. **a**–**e** Expansion STED (ExSTED). **a** Magnified view of the microtubules, showing clear distinction of the antibody positions on the opposing sites of a single microtubule. Scale bar, 100 nm. **b** Quantification of the apparent diameter of microtubule and the 2D resolution. **c** 3D STED of the expanded microtubules using an oil objective. Scale bar, 500 nm. **d** Model of the centrioles (blue) and the centriole-associated protein CEP152. **e** ExSTED HeLa cells with CEP152 staining. Scale bar, 200 nm. **f**–**h** Expansion SIM (ExSIM). **f** Sectioning of the expanded sample. **g** Z-projection of the reconstructed SIM image. **h** By matching the RI of the immersion oil with the sample, fine details of the *Drosophila* SC structure can be analyzed. Scale bars, 5 μm. **i**–**o** Expansion SMLM (ExSMLM). **i** dSTORM image of pre-labeled, proExM-expanded and re-embedded Cos-7 cells stained with primary antibodies against α-tubulin and Alexa Fluor 532-conjugated secondary antibodies. Scale bar, 500 nm. **j** 3D dSTORM image of the expanded and re-embedded Cos-7 cells labeled with anti α- and β-tubulin antibodies and additionally post-labeling with anti-α-tubulin. Scale bar, 5 μm. **k** Magnified view of highlighted region (white box) in **j**. Scale bar, 500 nm. **l** Image projection of the xz-axes averaged along two microtubule filaments shown in **j**. Scale bar, 100 nm. **m** Cross-sectional profile (blue dots) of the xz-projection shown in **l**. Using a bi-Gaussian fit (red), the peak-to-peak distance is determined to 81.2 nm. **n**, **o** 3D dSTORM image of U-ExM expanded and re-embedded Chlamydomonas centrioles stained post re-embedding. Zoom-in image of the boxed area in **n** is shown in **o**. Scale bar, 1 μm (**n**), 500 nm (**o**).   (**a**–**e**) Reprinted/adapted with permission from Gao et al. [97]. Copyright 2018 American Chemical Society. **f**–**h** Reprinted/adapted by permission from Springer Nature: Wang et al. [99], copyright 2018. **i**–**o** Reprinted/adapted from Zwettler et al. [43]

Samples can be imaged in its native aqueous environment in STED. This allows ExM hydrogels to stay in water (rather than a special buffer) during the imaging. In addition, immobilization of the ExM sample to the coverslips and/or the use of long working-distance objectives on a confocal setup can circumvent the imaging depth issue. Finally, the labeling density issue can be addressed by enhancing the labeling quality or applying multiple rounds of labeling. For example, three types of primary antibodies, including anti-GFP (to target the endogenously-expressed α-tubulin-GFP), anti-α-tubulin, and anti-β-tubulin, have been deployed to stain multiple epitopes of microtubules in HeLa cells in the ExSTED protocol (Fig. 16) [97]. This has resulted in significantly brighter microtubules for the STED imaging.

To determine the effective resolution of ExSTED, single imaging spots have been fitted with a 2D Gaussian, and line intensity profiles across the imaged microtubules have been fitted with a 1D Gaussian to determine their full width half maxima (FWHM). As a result, the effective lateral resolution of ExSTED has been determined to be ~ 8 nm from both validations. To test if ExSTED can perform sub-100 nm resolution imaging in both lateral and axial dimensions, 3D ExSTED has been applied to visualize the structure of microtubules. The final image has achieved an isotropic resolution of ~ 45 nm with negligible distortion by the expansion (< 1%) (Fig. 16). When the objective is switched from an oil-immersion objective (1.42 NA, 60×) to a water-immersion objective (1.2 NA, 60×) in order to increase the imaging depth, an isotropic resolution of ~ 70 nm has been achieved for ExSTED.

#### Expansion SIM (ExSIM)

Many of the challenges facing ExSTED also apply to SIM imaging of ExM samples [98, 99]. For example, the imageable depth of ExM sample is often limited by the short working distance of high-NA objectives used for conventional SIM imaging. Furthermore, reduced concentration of fluorescent labels leads to lower signals and faster photobleaching under SIM.

To overcome the working distance issue, a sample sectioning step has been introduced in one version of ExSIM (ExM-SIM) (Fig. 16) [99]. This physical sectioning process serves to reduce the sample thickness and so that each section can be imaged through. Using this protocol, images from each section are first obtained by SIM. These images are then aligned and averaged (as described below) to reconstruct the section and the entire sample. After expansion, however, the sample sectioning becomes more challenging due to the low mechanical instability of the gel. This issue has been solved by introducing a dehydration step with 30% (w/v) sucrose overnight before the sectioning.

To reduce the noise and improve the signal-to-background ratio, a strategy similar to single-particle averaging in cryogenic electron microscopy (cryo-EM) has been introduced in the ExM-SIM protocol [99]. In this protocol, classification and reconstruction of the images is relatively simpler than the cryo-EM averaging method because of the more explicit 3D information obtained by fluorescence microscopy. Briefly, appropriate single particles in the images are first selected. Next, different views as well as conformations of these particles are identified. The final image is then reconstructed after these views are subject to image averaging. Specifically, particles of the same conformational class, while different in the orientations, are combined into a high-resolution 3D image.

Using ExM-SIM, *Drosophila* synaptonemal complex (SC), a meiosis-specific multiprotein complex that facilitates crossing over between homologous chromosomes, has been imaged at a lateral resolution of ~ 30 nm (Fig. 16) [99]. In the ExM-SIM image, two distinct layers of the SC can be clearly seen. Moreover, one of the proteins in the middle of the SC, which typically appears as one single track through a conventional confocal microscope, can now be distinguished as two parallel tracks.

#### Expansion SMLM (ExSMLM)

Combining ExM and SMLM faces additional challenges on top of those described for ExSTED and ExSIM. For example, a photoswitching buffer (consisting of, for example, 100 mM mercaptoethylamine (MEA) in phosphate-buffered saline (PBS) with optimized pH, adjusted with KOH) is necessary for SMLM imaging. The ionic content of the buffer can shrink the size of the gel. In addition, organic dyes commonly used for SMLM (e.g., Alex Fluor 647) are often not compatible with free-radical chain-growth polymerization, and thus cannot be used for pre-expansion staining of the sample.

To reduce the buffer effect, the sample-hydrogel composite can be re-embedded in a non-expandable hydrogel, similar to the aforementioned iterative expansion workflow (Fig. 14). As a result, the buffer no longer shrinks the sample, as the size of the non-expanding hydrogel does not get affected by the ionic concentration (i.e., the non-expanding gel locks the sample to its expanded state).

The aforementioned dye degradation issue can be circumvented by using post-expansion immunostaining so that the antibody-conjugated dyes do not get destroyed during the polymerization [43]. As a result, combining ExM and dSTORM (one of the SMLM techniques) can achieve ~ 15–20 nm or even better spatial resolution (Fig. 16).

Finally, like STED and SIM, high-magnification, high-NA oil-immersion objectives commonly used for SMLM (under the Total Internal Reflection Fluorescence (TIRF) mode) only reach a limited distance from the coverslip surface. To circumvent this issue, long working-distance water-immersion objectives (for epifluorescence illumination) [43] and water-dipping objectives (for light-sheet illumination) [100] have been used for ExSMLM.

### Labeling size and density

In ExM, target molecules and fluorescent labels are volumetrically diluted due to the expansion process. Additionally, a good portion of the target molecules and labels can be lost after the expansion due to the low anchoring efficiency and/or the removal of these molecular species during the homogenization and expansion step. Dilution or loss of the molecules and labels leads to reduced signal-to-background ratio and punctation of the biological structures of interest, which can substantially degrade the quality (and the attainable resolution) of ExM imaging. Finally, the physical size of the molecular probes can introduce additional spatial errors to the locations of the anchored biomolecules in the ExM images.

In this section, we will review ExM methods and protocols that address the issue of labeling density and accuracy. One strategy that has been extensively explored in the field is to preserve the target molecules better (e.g., to better retain the epitopes) and/or amplify the fluorescent labels (either at the pre-expansion or post-expansion states). In the previous section (see Sect. 5.2), we have described some of its early implementations. In ExSTED, for example, the labeling dilution issue has been addressed by applying multiple rounds of primary antibody staining (i.e., by increasing amount of the fluorescent dye) against the same protein [97]. Another strategy is to develop smaller molecular probes. Aside from the reduced labeling size effect, smaller probes can diffuse through the sample faster and conjugate to their targets more efficiently, which may also ameliorate the labeling density issue.

#### Increasing the labeling density

Loss of the fluorescent labeling often happens in the multi-round expansion protocol because the anchored molecules and fluorescent tags can detach from the hydrogel backbones when their linkage with the first-round gel breaks and another linkage with the second-round gel forms. In pan-ExM, for example, a new method called semi-interpenetrating polymer networks (semi-IPNs) has been introduced to circumvent this issue [47]. The semi-IPNs strategy enables direct linkage between the target molecules and the polymer network of the second-round gel (without breaking the linkage to the first-round gel). As a result, potential protein loss during the linkage switch is reduced. After additional validations and control experiments, the polymer entanglement (rather than the crosslinking between the first-round and the second-round expanding gels) has been validated to play an important role in the observed protein retention.

Increasing the specificity and efficiency of the fluorescent labeling (at the post-expansion state) is another promising strategy to increase the labeling density for ExM. The ExR and decrowding pathology (dExPath) protocols, for example, increase the immunostaining efficiency by creating additional space around the anchored proteins and potentially making more binding accessible to the antibodies [52, 101]. Compared with conventional ExM protocols with antigen retrieval, the ExR protocol, for example, has revealed more structural details for synapse proteins, including the visualization of ultrastructure of calcium channels, RIM1/2, PSD95, SynGAP, and Bassoon (Fig. 15). ExR protocol has also been used to reveal the 3D shapes and the periodic structures of Aβ42 and Kv7.2 puncta in the Alzheimer’s disease mouse brains (Fig. 15).

#### Reducing the labeling size

As described earlier, pre-expansion immunostaining is a common labeling strategy for proExM. However, primary and secondary antibodies can be as large as ~ 15–20 nm when combined, and the spatial errors introduced by the antibodies will be enhanced by the subsequent expansion step. Particularly, this labeling size effect becomes an issue when the target resolution is at the length scale of an antibody or below. In this case, either a molecular probe with a smaller size or a post-expansion labeling strategy [42–44] is preferred.

In label-retention ExM (LR-ExM), protein tags, such as SNAP and CLIP tags, have been used to reduce the label size [55]. These genetically encoded tags are small in size (~ 2 nm), and can be conjugated to the target proteins and then fluorescently labeled with a highly specific ligand. To anchor the protein tags to the hydrogel, trifunctional anchors, such as benzylguanine (BG)-methacrylamide (MA)-biotin (for SNAP tags) and benzylcytosine (BC)-MA-biotin (for CLIP tags), have been synthesized. In this anchor design, the BG and BC groups react specifically with the protein tags (i.e., serving as the ligands), while the MA group links the anchor to the gel. The biotin group is designed to bind fluorescent reporters.

A similar trifunctional linker (e.g., NHS-MA-biotin) renders LR-ExM compatible with primary antibody staining (without the need for secondary antibodies). This further increases the utility of LR-ExM while keeping the size of the labeling as small as a primary antibody (~ 7–10 nm). Finally, LR-ExM has been combined with super-resolution imaging methods, such as SIM and STORM. For example, LR-ExSIM has been used to visualize primary antibody-labeled distal appendages in the primary cilia of retinal pigment epithelium cells. Using these structures, the final resolution of LR-ExSIM and LR-ExSTORM has been determined to be ~ 34 nm and ~ 2 nm, respectively (Fig. 15).

### Expansion isotropy

Contrary to the existing super-resolution microscopy methods, ExM improves the effective imaging resolution by enlarging the physical size of the biologic sample. However, distortion and degradation of the original spatial information can occur during the expansion process, which will lead to an inaccurate representation of the molecular arrangements after the expansion.

Commonly used ExM hydrogels, including the polyacrylamide/sodium polyacrylate gel, are synthesized via free-radical chain-growth polymerization, which is known to introduce nanoscopic heterogeneities to the polymer network at the nanometer to tens-of-nanometer length scale [102–106]. In addition, topological defects of the polymer chains, such as loops and dangling ends, are known to introduce additional  structural inhomogeneities at the nanometer length scale to the gel.

All these structural imperfections existing within the expansion matrix can lead to a potential distortion of the post-expansion molecular arrangements compared to its pre-expansion state, which will make the interpretation of the final images challenging. To make things worse, these issues cannot be mitigated by changing the labeling strategies or the sample preservation methods as they arise from the intrinsic chemical and physical properties of the expansion matrix. In order to achieve nanoscopic resolution in its full sense, the local distortion of the ExM hydrogel matrix needs to be addressed.

In this section, we will review the recent progress in addressing the intrinsic limitation of expansion isotropy. Specifically, we describe the on-going development of structurally homogeneous hydrogels that allows highly isotropic expansion.

#### Hydrogel matrix for high-isotropy expansion

Conventional ExM hydrogels, including the polyacrylamide/sodium polyacrylate gel and the poly(*N,N*-dimethylacrylamide)/sodium polyacrylate gel (DMAA gel), suffer from structural anisotropy in their polymer network [102–106]. This anisotropy arises from (a) spatial fluctuation of monomers and cross-linking densities at the ~ 15–25 nm length scale, and (b) dangling polymer chains and (c) formation of polymer chain loops at the ~ 1–10 nm length scale. As described earlier, structural anisotropy of the hydrogel network can lead to local expansion error and distorted representation of the original molecular arrangements in ExM.

**Fig. 17:**
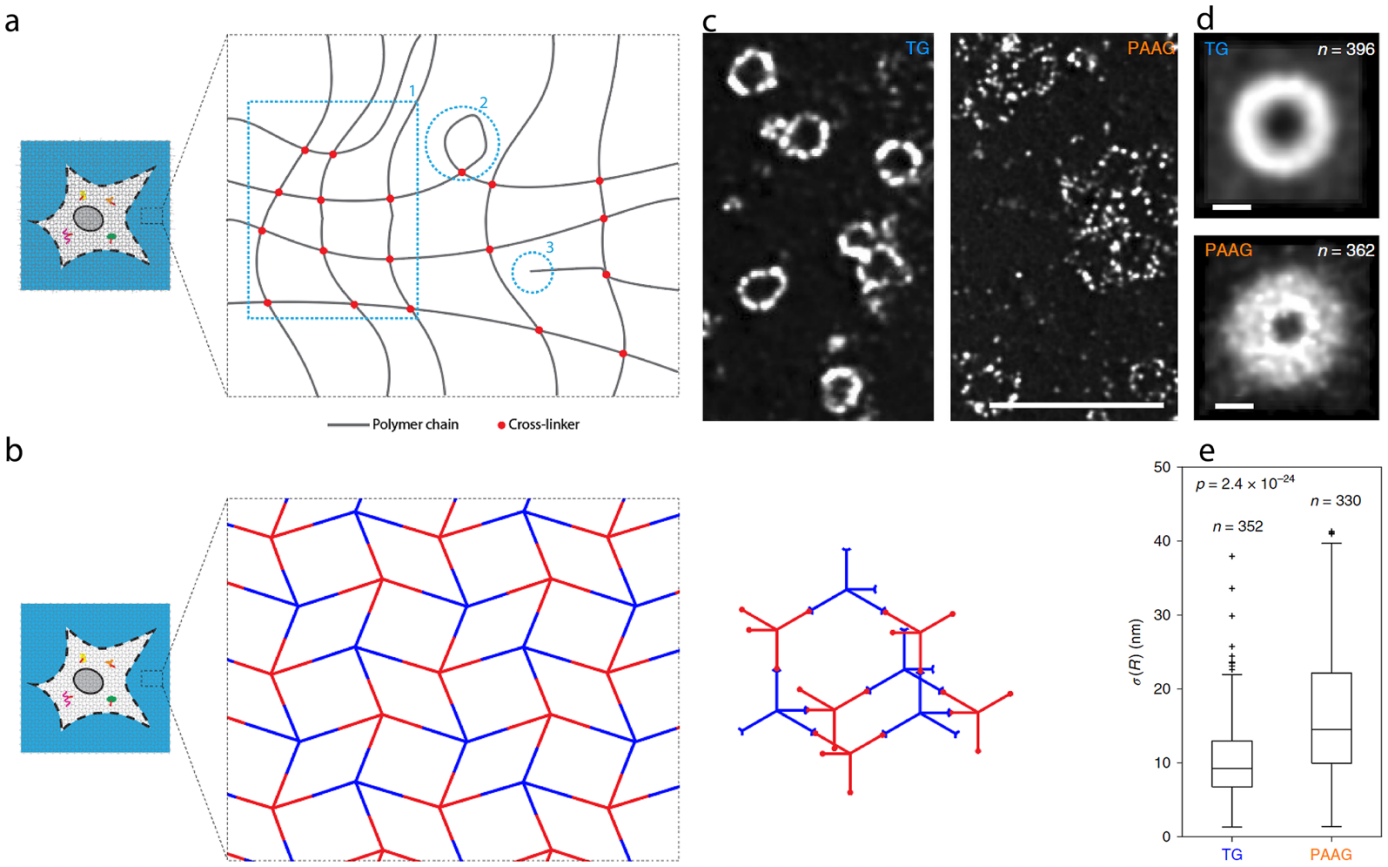
Chemical design and imaging validation of tetra-gel (TG)-ExM for high-isotropy expansion.** a** Possible structural defects of hydrogels synthesized via free-radical chain-growth polymerization. **b** Chemical components of TG. **c** HSV-1 virions with directly labelled envelope proteins, expanded by TG- (left) and PAAG-based (right) two-round iterative expansion. Scale bar (for both panels), 1 μm (TG, 10.3 μm; PAAG, 15.3 μm). **d** Single-particle averaged images of HSV-1 virions after TG- (top) and PAAG-based (bottom) two-round iterative expansion (TG, n = 396; PAAG, n = 362 virions). Scale bars, 100 nm. **e** Standard deviation (*σ*) of HSV-1 virion radius (*R*) within individual virions for TG- and PAAG-based iterative expansion (*p* = 2.4 × 10^−24^, two-sided Wilcoxon rank sum test; TG, n = 352; PAAG, n = 330 virions). (**c**–**e** Reprinted/adapted by permission from Springer Nature: Gao et al. [54], copyright 2021)

A new hydrogel called tetra-gel (TG) has recently been designed to mitigate this structural anisotropy. Instead of free-radical chain-growth polymerization, TG polymerizes via non-radical, click chemistry-based step-growth polymerization [53, 54]. Specifically, TG is synthesized via terminal linking of two complementary tetrahedral monomers—one charged and one neutral—that form a diamond lattice-like polymer network (Fig. 17). By design, TG network does not have dangling polymer chains or loops, and the distribution of the TG monomers is uniform throughout the gel.

To evaluate TG’s structural homogeneity and its expansion accuracy, a spherical virus HSV-1 is iteratively expanded using TG as the first-round expanding gel (while using the polyacrylamide/sodium polyacrylate gel as the second-round expanding gel) (Fig. 17). Prior to the expansion, the virion envelope proteins are directly labeled with a small oligo (~ 7 nm) to reduce the labeling size. Using this direct labeling, the spatial information of the envelope proteins is encoded at the location of the oligos, which can be transferred across the gels during the subsequent iterative expansion process, similar to the iExM protocol.

As a result, the spatial errors associated with the TG (~ 9.2 nm) has been determined to be significantly smaller than that of the polyacrylamide/sodium polyacrylate gel (~ 14.3 nm) from both the averaged particle and ensemble analyses [54]. In addition, TG has been validated to significantly better preserve the (spherical) shape of the virus particle compared to the polyacrylamide/sodium polyacrylate gel. Furthermore, a study using DNA origami has found that TG better preserves periodic nanostructures than the polyacrylamide/sodium polyacrylate gel, with former and latter introducing a ~ 5 and ~ 16 nm standard deviation in the spatial displacements, respectively [53]. Combined, these results suggest that TG can better preserve the nanoscopic molecular arrangements in the sample compared to the conventional ExM gels.

## Conclusions and perspectives

Recent development of ExM has enabled nanoscale fluorescence imaging of biological structures and molecular compositions in preserved biological samples with no or minimal need to change the optics system and the post-imaging computational pipeline. The relatively straightforward sample preparation and its high compatibility with conventional fluorescence labeling techniques has rendered ExM applicable to a wide range of biological and clinical investigations. Importantly, the continuing effort to increase its expansion factor and to combine it with better sample preservation methods [107] is rapidly expanding ExM’s technological boundary and broadening its applicability in nanoimaging.

Future development of ExM methods is, however, not without challenges. Some of the inherent limitations of the method and the early attempts to overcome these limitations have already been described (Sect. 5). For example, the issue of low labeling density will continue to limit the achievable spatial resolution of ExM. In particular, the inadequate labeling can cause significant underrepresentation of the target structures and molecules. While it poses the same challenge to all super-resolution microscopy methods, this effect is prominent in ExM because the labeling density decreases volumetrically with the expansion. The recent reports of decrowding the target molecules and improving the labeling efficiency and specificity provides a potential path towards high-density post-expansion labeling in ExM [52]. Nevertheless, continuing efforts are necessary to enhance the sample labeling density for both the pre- and post-expansion labeling processes.

As ExM becomes compatible with more biomolecules and chemical species owing to the development of novel labeling and anchoring chemistries, the number of target molecules in single imaging session will increase drastically. This in turn raises a challenge for ExM because the number of fluorescent color channels is limited in a single round of imaging. Recent reports have adopted spectral mixing [108] and multi-round fluorescence imaging [89, 109, 110] to address this issue. As the molecular multiplexibility further increases, the development of a fast, multi-channel, and scalable imaging modality has become an urgent task of the field.

Finally, expansion isotropy (or expansion accuracy), a measurement of how faithful the relative positions of the anchored molecules and fluorescent tags are be preserved after the expansion, will continue to be an important factor that limits the final effective resolution of ExM. One recent attempt to improve the expansion isotropy has proposed to switch the expansion matrix to a more structurally homogenous hydrogel composed of tetrahedral monomers [54]. This raises an intriguing question of what the best polymer network topology [106], monomer design [111], and polymerization mechanism [112–114] will be in order to further improve the method’s resolution and accuracy. Additionally, the spatial errors associated with the size of the labeling need to be minimized to achieve a better accuracy. This may involve development of smaller probes, such as nanobodies and aptamers, as well as a direct modification and labeling strategy for endogenous biomolecules. It remains to be answered whether such efforts can push the isotropy and accuracy of the expansion process to the single-molecule level.

## Data Availability

The review is based on the published data and sources of data upon which conclusions have been drawn can be found in the reference list.
